# Comparative analysis of carotenoid accumulation in two goji (*Lycium barbarum* L. and *L. ruthenicum* Murr.) fruits

**DOI:** 10.1186/s12870-014-0269-4

**Published:** 2014-12-16

**Authors:** Yongliang Liu, Shaohua Zeng, Wei Sun, Min Wu, Weiming Hu, Xiaofei Shen, Ying Wang

**Affiliations:** Key Laboratory of Plant Germplasm Enhancement and Specialty Agriculture, Wuhan Botanical Garden, Chinese Academy of Sciences, Wuhan, Hubei 430074 China; Key Laboratory of Plant Resources Conservation and Sustainable Utilization, South China Botanical Garden, Chinese Academy of Sciences, Guangzhou, Guangdong 510650 China; Northwest Center for Agrobiotechnology (Ningxia), Chinese Academy of Sciences, Beijing, China; Institute of Chinese Materia Medica, China Academy of Chinese Medical Sciences, Beijing, 100700 China; University of the Chinese Academy of Sciences, Beijing, 100049 China

**Keywords:** Carotenoids, Chromoplast, Fruit development, Gene expression, *Lycium barbarum*, *L. ruthenicum*

## Abstract

**Background:**

The traditional Chinese medicinal plants *Lycium barbarum* L. and *L. ruthenicum* Murr. are valued for the abundance of bioactive carotenoids and anthocyanins in their fruits, respectively. However, the cellular and molecular mechanisms contributing to their species-specific bioactive profiles remain poorly understood.

**Results:**

In this study, the red fruit (RF) of *L. barbarum* was found to accumulate high levels of carotenoids (primarily zeaxanthin), while they were undetectable in the black fruit (BF) of *L. ruthenicum*. Cytological and gene transcriptional analyses revealed that the chromoplast differentiation that occurs in the chloroplast during fruit ripening only occurs in RF, indicating that the lack of chromoplast biogenesis in BF leads to no sink for carotenoid storage and the failure to synthesize carotenoids. Similar enzyme activities of phytoene synthase 1 (PSY1), chromoplast-specific lycopene β-cyclase (CYC-B) and β-carotene hydroxylase 2 (CRTR-B2) were observed in both *L. ruthenicum* and *L. barbarum*, suggesting that the undetectable carotenoid levels in BF were not due to the inactivation of carotenoid biosynthetic enzymes. The transcript levels of the carotenoid biosynthetic genes, particularly *PSY1*, *phytoene desaturase* (*PDS*), *ζ-carotene desaturase* (*ZDS*), *CYC-B* and *CRTR-B2*, were greatly increased during RF ripening, indicating increased zeaxanthin biosynthesis. Additionally, *carotenoid cleavage dioxygenase* 4 (*CCD4*) was expressed at much higher levels in BF than in RF, suggesting continuous carotenoid degradation in BF.

**Conclusions:**

The failure of the chromoplast development in BF causes low carotenoid biosynthesis levels and continuous carotenoid degradation, which ultimately leads to undetectable carotenoid levels in ripe BF. In contrast, the successful chromoplast biogenesis in RF furnishes the sink necessary for carotenoid storage. Based on this observation, the abundant zeaxanthin accumulation in RF is primarily determined via both the large carotenoid biosynthesis levels and the lack of carotenoid degradation, which are regulated at the transcriptional level.

**Electronic supplementary material:**

The online version of this article (doi:10.1186/s12870-014-0269-4) contains supplementary material, which is available to authorized users.

## Background

Carotenoids are isoprenoids that are synthesized by all photosynthetic organisms as well as some non-photosynthetic bacteria and fungi. In plants, chloroplastic carotenoids are constituents of light-harvesting complexes and the photosynthetic reaction center, where they also play important roles in protecting tissues against photo-oxidative damage [1,2]. When accumulated in the chromoplasts of flowers and fruits, carotenoids act as visual attractants for pollinating insects and seed-dispersing animals [3,4]. Furthermore, carotenoids are the precursors of important apocarotenoids, such as volatile flavor/aroma terpenes, and the growth regulators abscisic acid (ABA) and strigolactone [5-7]. Recently, oxidized products from plant carotenoids have been implicated as signals induced by environmental stressors [8]. In addition to these biological functions, carotenoids serve as major micronutrients in the human diet [9,10]. In particular, β-carotene, α-carotene and β-cryptoxanthin are precursors for vitamin A biosynthesis [11], while lutein and zeaxanthin slow aging-related damage to the retina [12].

During the past two decades, the carotenoid biosynthetic pathway in plants has been well elucidated [13-15]. Previous studies have shown that there are at least three mechanisms that regulate carotenoid accumulation in the chromoplasts [16]. First, the transcript abundance of rate-limiting structural genes is predicted to be the primary mechanism controlling the carotenoid content and composition in the chromoplasts [17]. During tomato (*Solanum lycopersicum*) fruit development, increasing expression of *phytoene synthase 1* (*PSY1*) and *phytoene desaturase* (*PDS*), diminishing expression of the chloroplast-related *lycopene β-cyclase* (*LCY-B*) and *lycopene ε-cyclase* (*LCY-E*), and low transcript levels of the chromoplast-specific *LCY-B* and *β-ring hydroxylase* (*CRTR-B*) (corresponding to *CYC-B* and *CRTR-B2*, respectively), lead to the accumulation of lycopene as the major carotenoid [18-21].

Second, carotenoid degradation by carotenoid cleavage dioxygenases (CCDs) may be central to determining the final carotenoid concentrations in chromoplasts [22]. For example, despite active carotenoid biosynthesis in both the yellow and white petals of chrysanthemums (*Chrysanthemum morifolium*), the carotenoids are degraded by CmCCD4a into colorless compounds in the white petals [23]. In potatoes and peaches, the different carotenoid content among the cultivars can also be attributed to the distinct enzymatic activity of the CCD4-degrading carotenoids [24-26].

Finally, the sink capacity of carotenoid-accumulating tissues has recently been implicated in the control of carotenoid levels. The characterization of the Orange (Or) protein, which is involved in chromoplast biogenesis, revealed the importance of the carotenoid storage sink for carotenoid accumulation. Due to a failure in chromoplast formation, the cauliflower (*Brassica oleracea*) *Or* mutant lacks carotenoid accumulation [27]. When the *Or* gene was transformed into Arabidopsis, the Arabidopsis calli exhibited an orange color with chromoplast formation [28]. In tomatoes, the perturbed activity of several light-signal-related genes, including *UV-Damaged DNA Binding Protein1* (*DDB1*) [29], *De-Etiolated1* (*DET1*) [30,31], *Cullin4* (*CUL4*) [32], *HY5*, *COP1LIKE* [33], *Cryptochrome 2* (*CRY2*) [34], *Golden 2-Like* (*GLK2*) [35] and *Arabidopsis Pseudo Response Regulator2-Like* (*APRR2-Like*) [36], caused changes in the plastid number and size, which indirectly affected the concentrations of the carotenoids and other phytonutrients in the ripening fruits [37].

*Lycium barbarum* L. (Chinese: gouqi or ningxiagouqi) and *L. ruthenicum* Murr. (Chinese: heigouqi or heiguogouqi), which are two shrub plants belonging to the Solanaceae family, have been used as traditional medicinal plants in China and other Asian countries for centuries [38]. *L. barbarum*, in particular, has high economic significance in Northwest China, with its red fruit (RF; gouqizi in Chinese, also known as goji berry or wolfberry) being used for both traditional Chinese medicine (TCM) and nutritional purposes [39]. Modern pharmacological studies have begun to investigate the biochemical mechanisms of the medicinal effects of wolfberry, including the antioxidant, immunomodulatory and neuroprotective properties, which are primarily attributed to the polysaccharides (LBP), flavonoids and carotenoids [40,41]. *L. ruthenicum* is another TCM used for the treatment of heart disease, abnormal menstruation and menopause [42]. The functional compounds in the black fruit (BF) of *L. ruthenicum* are primarily comprised of anthocyanins, essential oils and polysaccharides [42-45]. As the primary pigment in RF, carotenoids have been extensively studied, and zeaxanthin and esterified zeaxanthin were reported to be the major bioactive compounds that accumulate in RF, especially for its traditional use in eyesight improvement [41,46]. However, the content and composition of the carotenoids in BF have not been comprehensively reported, and the mechanisms controlling the species differences in the carotenoid biosynthesis between RF and BF remain unknown. Analyses of these differences may provide novel insights into the regulation of carotenoid accumulation in goji fruits, with important implications for their medicinal and nutritional value.

## Results

### The carotenoid accumulation differs between the RF and BF from different *Lycium* species

**Figure 1:**
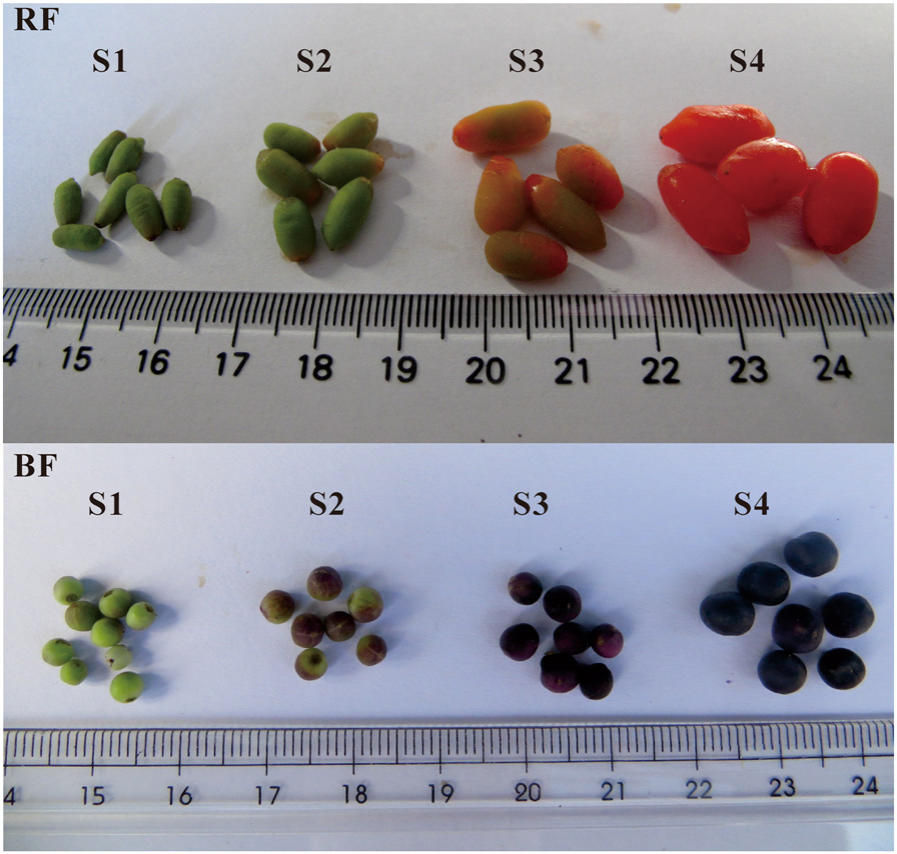
**Photographs of** ***L. barbarum*** **and** ***L. ruthenicum*** **fruits (RF and BF, respectively) at different developmental stages (S1-S4).** S1, green fruit stage; S2, color break stage; S3, light color stage; S4, ripe fruit stage.

The analysis of the total carotenoid content in the red fruits of *L. barbarum* and the black fruits of *L. barbarum* at four developmental stages (S1-S4, Figure 1) revealed an increase in the carotenoid content of RF from S2 to S4, reaching a maximum of 508.90 μg g^−1^ fresh weight (FW) (Additional file 1). On the converse, the amount of carotenoids in BF declined from 34.46 μg g^−1^ FW in the S1 fruit to undetectable levels in the S4 fruit (Additional file 1).

**Figure 2:**
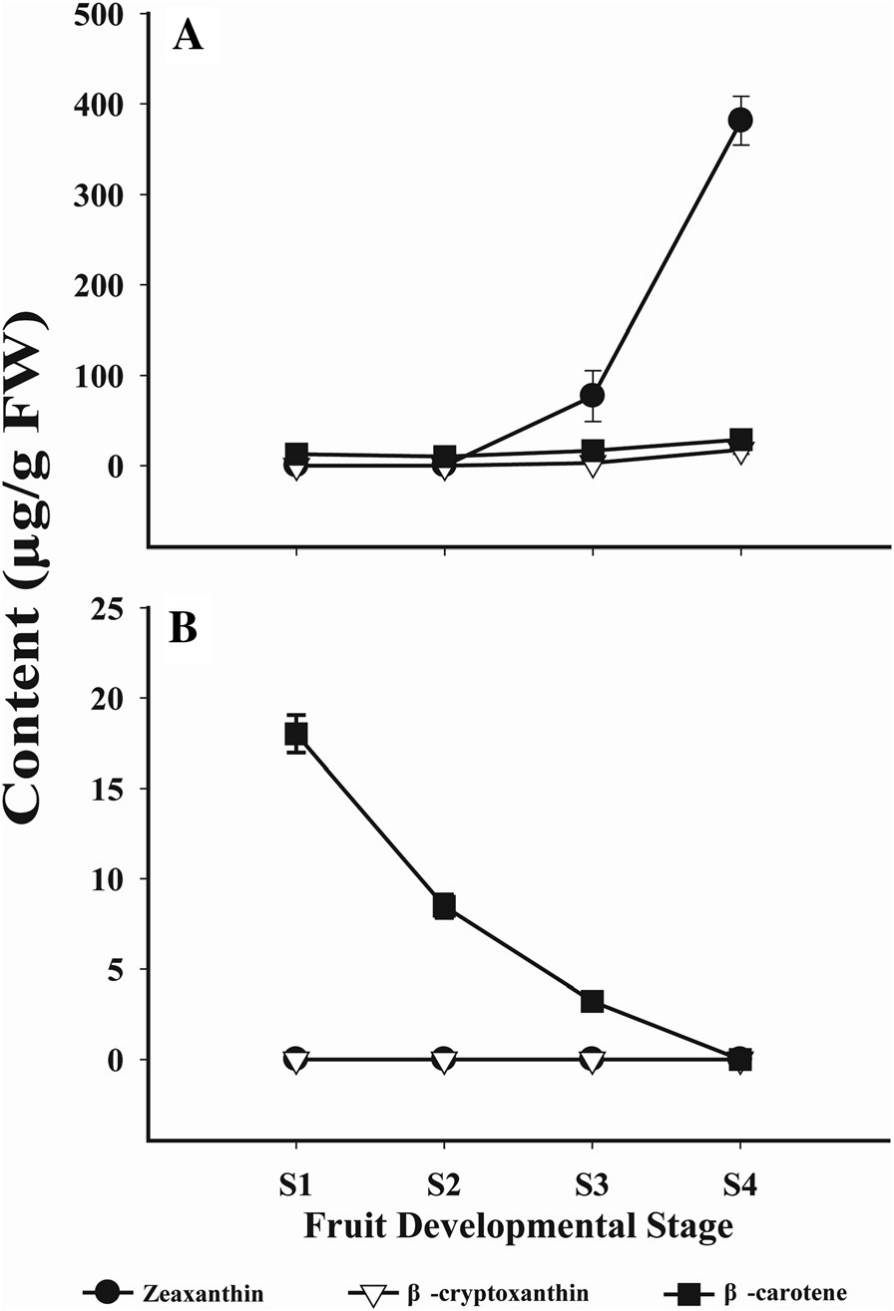
**Content of zeaxanthin, β-cryptoxanthin and β-carotene in RF (A) and BF (B).**

The carotenoid composition and content in ripe RF were previously reported [46], with zeaxanthin accounting for the highest carotenoid proportion, followed by β-cryptoxanthin and β-carotene, and with most of the xanthophylls esterified. In this study, to detect the carotenoid accumulation regardless of esterification, the carotenoid content in the four developmental stages (S1-S4, Figure 1) of RF and BF was analyzed after saponification. Xanthophyll esters were undetectable in BF (data not shown). In S1 of RF and BF, the chloroplastic carotenoids, violaxanthin, lutein and β-carotene comprised the majority of the carotenoids (Additional files 2 and 3). Two additional compounds (both unidentified) were detected in BF. As both the red and black fruits developed, the amount of chloroplastic carotenoids (lutein, violaxanthin and β-carotene) declined (Additional files 2 and 3). During BF ripening, no additional carotenoids showed rising levels, and all of the existing carotenoids gradually decreased to undetectable levels (Figure 2B; Additional files 1 and 3). Meanwhile, in RF, several other carotenoids, especially zeaxanthin, increased dramatically from S2 to S4 (Figure 2A; Additional files 1 and 2). Specifically, zeaxanthin reached 381.6 μg g^−1^ FW, and β-cryptoxanthin and β-carotene reached 17.59 μg g^−1^ FW and 28.99 μg g^−1^ FW, respectively (Additional file 1).

### Light microscopy of the green fruits (S1) and ripe fruits (S4)

**Figure 3:**
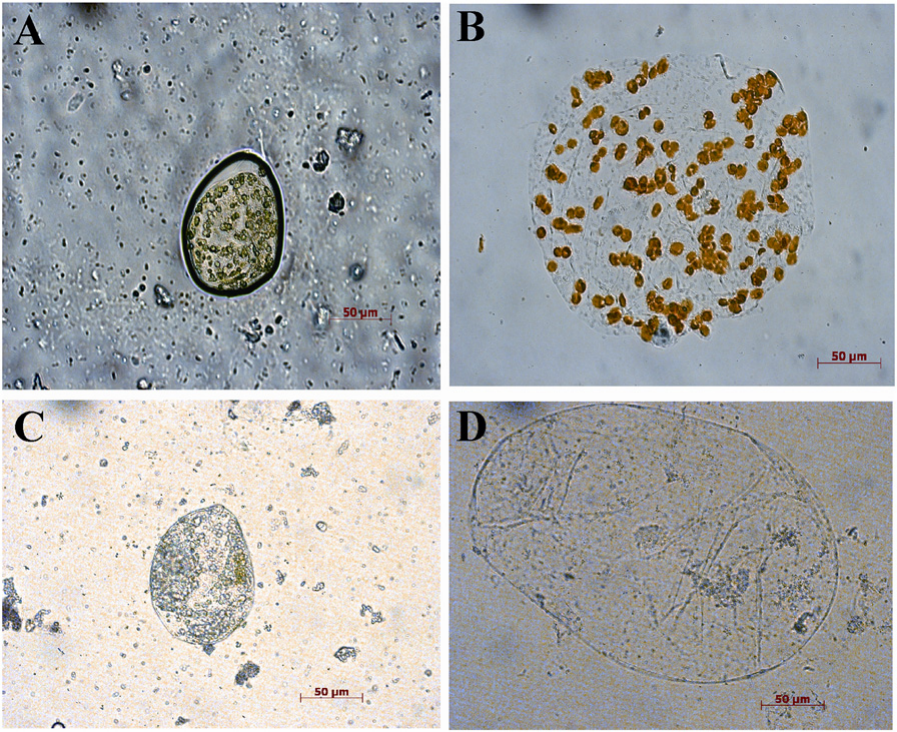
**Light micrographs of plastids in RF and BF. (A)** Green RF (S1) cell with chloroplasts. **(B)** Ripe RF (S4) cell with orange globular chromoplasts. **(C)** Green BF (S1) cell with chloroplasts. **(D)** Ripe BF (S4) cell without colour chromoplasts. Fruits are not stained to show the natural colour of plastids.

The chromoplast differentiation in the fruits of the two *Lycium* species was comparatively determined by examining the plastids in the mesocarp of the green fruits (S1) and the ripe fruits (S4) under a light microscope. The chloroplasts were observed in the green fruits of both species (Figures 3A and C). However, orange, globular chromoplasts were only observed in the ripe RF of *L. barbarum* (Figure 3B). Consistent with the absence of carotenoid accumulation, a failure in chromoplast formation was observed in the ripe BF of *L. ruthenicum* (Figure 3D).

### Isolation of the carotenogenesis-related genes from *L. barbarum* and *L. ruthenicum*

To compare the gene sequences encoding the enzymes responsible for the biosynthesis, degradation, and storage of carotenoids in both species, the full-length open reading frames (ORF) of twenty-five putative carotenogenesis-related genes were isolated (Table 1). The phylogenetic relationship of each putative protein (with known functional proteins in other organisms) was used to confirm the orthologous relationships of these proteins with the clearly defined proteins in tomatoes (Additional file 4). Two isoforms of *1-deoxy-D-xylulose 5-phosphate synthase* (*DXS1* and *DXS2*), which are involved in the first step of the 2-*C*-methyl-Derythritol4-phosphate (MEP) pathway, were isolated in each species. In particular, three pairs of carotenoid biosynthetic genes (*PSY1*/*PSY2*, *LCY-B*/*CYC-B*, *CRTR-B1*/*CRTR-B2*) were isolated; *PSY1*, *CYC-B* and *CRTR-B2* are putatively specific for carotenoid biosynthesis in chromoplasts [18]. The ORF length of the majority of the genes [except for *15-cis-ζ-carotene isomerase* (*Z-ISO*), P450-type *ε-ring hydroxylase* (*CYP97C11*), *CRTR-B1* and *zeaxanthin epoxidase* (*ZEP*)] was identical between the two *Lycium* species. The average identity of the protein sequences between *L. barbarum* and *L. ruthenicum* was 98.42%, while *L. barbarum* and *S. lycopersicum* shared 89.03% identity and *L. ruthenicum* and *S. lycopersicum* shared 88.83% identity. Consistent with other species, most of the proteins were predicted to localize to the chloroplast by ProtComp (Table 1).

**Table 1:** **Sequence information of the carotenogenesis-related genes from** ***L. barbarum*** **and** ***L. ruthenicum***

**Gene**	**ORF length**	**Protein length**	**cTP** ^**b**^	**Protein identity**	**Protein identity Lb vs Sl** ^**c**^	**Protein identity**
	**(bp** ^**a**^ **, Lb/Lr)**	**(Lb/Lr)**		**Lb vs Lr (%)**	**(accession number of Sl)** ^**d**^	**Lr vs Sl**
*DXS1*	2154/2154	717/717	YES	98.61	95.13 (FN424051)	95.13
*DXS2*	2139/2139	712/712	YES	98.88	92.30 (FN424052)	92.72
*PSY1*	1239/1239	412/412	YES	99.03	87.26 (ABU40772)	86.54
*PSY2*	1323/1323	440/440	YES	99.55	91.14 (ABU40771)	90.68
*PDS*	1749/1749	582/582	YES	98.28	93.83 (AGO05926)	94.17
*Z-ISO*	**1122/1131**	373/376	NO	97.07	86.90 (XP_004252966)	85.94
*ZDS*	1767/1767	588/588	YES	99.32	95.24 (AGO05927)	95.24
*CRTISO*	1815/1815	604/604	NO	99.67	93.33 (AAL91366)	93.01
*LCY-B*	1506/1506	501/501	YES	99.20	87.43 (NP_001234226)	87.43
*CYC-B*	1497/1497	498/498	YES	97.59	87.95 (*AAG21133*)	88.55
*LCY-E*	1572/1572	523/523	YES	99.62	92.41 (Y14387)	92.60
*CYP97A29*	1818/1812	605/603	YES	97.69	90.97 (ACJ25969)	89.98
*CYP97C11*	**1644/1641**	547/546	YES	98.54	91.64 (ACJ25968)	91.64
*CRTR-B1*	**915/906**	304/301	NO	98.36	87.38 (CAB55625)	85.76
*CRTR-B2*	939/939	312/312	NO	97.76	85.99 (CAB55626)	84.08
*ZEP*	**1989/1986**	662/661	YES	98.49	89.99 (P93236)	89.39
*VDE*	1413/1413	470/470	YES	98.30	88.49 (ACM92036)	88.49
*NCED1*	1824/1824	607/607	YES	99.01	89.22 (CAB10168)	88.56
*NCED6*	1764/1764	587/587	YES	96.08	80.74 (XP_004240215)	80.41
*CCD1A*	1623/1623	540/540	YES	98.89	93.03 (AAT68187)	93.76
*CCD4*	1800/1800	599/599	YES	99.83	85.86 (XP_004246004)	85.86
*CHRC*	966/966	321/321	YES	96.26	84.76 (ABC42191)	85.37
*Or1*	906/906	301/301	NO	99.34		86.90
*Or2*	945/945	314/314	NO	97.77		88.22
*HSP21*	711/711	236/236	YES	97.46	79.41 (AAB07023)	80.25
Average				98.42	89.03	88.83

^a^The size in base pairs of the putative coding region from the predicted ATG to the stop codon, and bold numbers indicate that the length of coding regions are different between *L. barbarum* (Lb) and *L. ruthenicum* (Lr); ^b^Softberry ProtComp (http://linux1.softberry.com/berry.phtml?topic=protcomppl&group=programs&subgroup=proloc) prediction for a chloroplast transit peptide; ^c^ *Solanum lycopersicum* (Sl); ^d^Accession from NCBI (http://www.ncbi.nlm.nih.gov/).

### The comparative RNA-seq profile of the carotenogenesis-related genes in the ripening fruits of the two *Lycium* species

**Figure 4:**
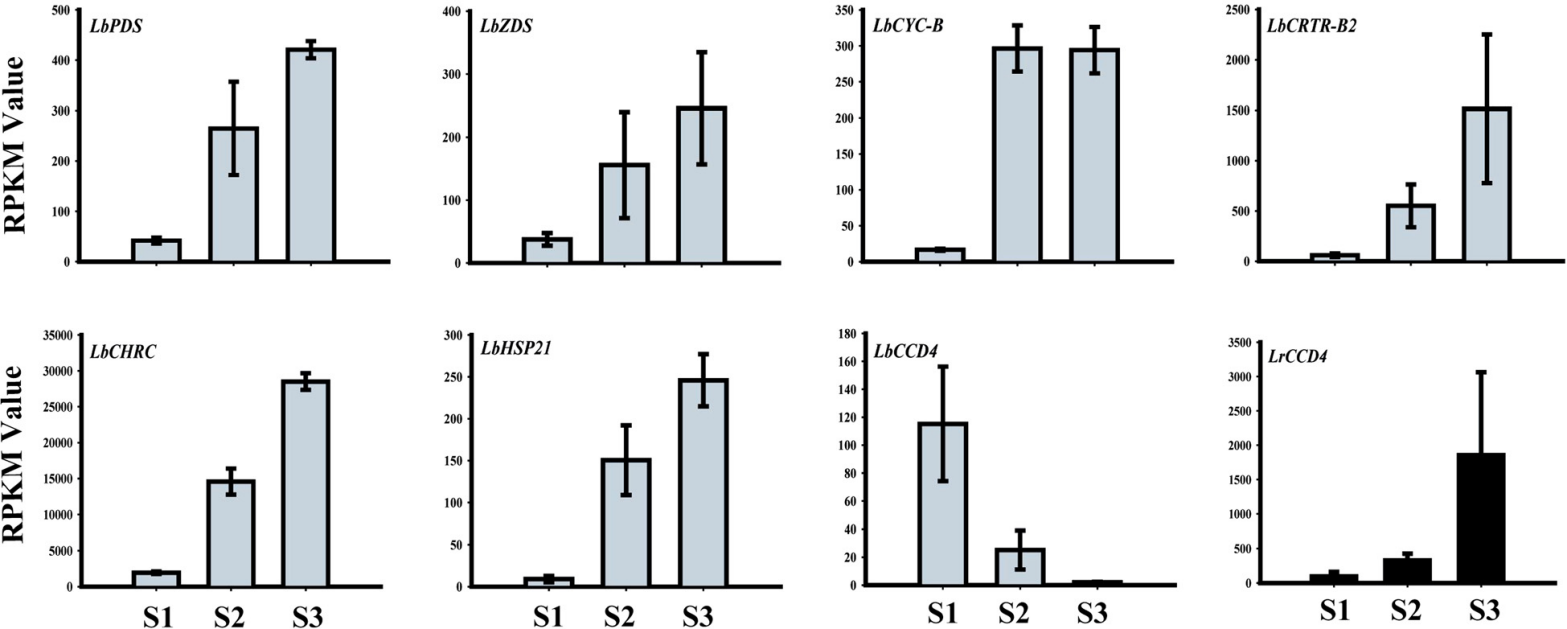
**The RPKM values, calculating from the RNA-seq data of** ***L. barbarum*** **and** ***L. ruthenicum*** **, of eight carotenoid-ralated genes which are obviously changing during fruit development.** *LrCCD4*, *carotenoid cleavage dioxygenase 4* from *L. ruthenicum*. Other seven genes are from *L. barbarum*: *PDS, phytoene desaturase; ZDS, ζ-carotene desaturase; CYC-B, chromoplast-specific lycopene β-cyclase; CRTR-B2, non-heme di-iron carotenoid β-ring hydroxylase 2; CHRC, Chromoplast-specific carotenoid-associated protein; HSP21, heat shock protein 21*; *CCD4*, *carotenoid cleavage dioxygenase 4*.

To comparatively overview the expression of the carotenogenesis-related genes in both species, RNA-seq data derived from the fruit’s S1 to S3 stages were profiled in this study. Generally, the parameters, transcriptional read amounts and reads per kilobase of coding sequence per million reads (RPKM) are used for assessing the gene expression levels when analyzing RNA-seq data. As shown in Additional file 5, the RPKMs of the 25 carotenogenesis-related genes were calculated. The RPKM of the chromoplast-related genes (*CHRC*, *Or1* and *HSP21*) in RF were much higher than those in BF, suggesting that these genes are more active in RF than in BF. Particularly, the RPKM of *CHRC* reached nearly 30,000 in RF (Figure 4). In RF, the RPKMs of some of the carotenoid biosynthetic genes (*PDS*, *ZDS*, *CYC-B* and *CRTR-B2*) showed increasing trends during fruit ripening and approached the hundreds in S2 and S3 (Figure 4, Additional file 5). In contrast, in all three BF stages, the RPKMs of all of the carotenoid biosynthetic genes were less than fifty (Additional file 5). During BF development, only the *LrCCD4* transcripts increased and sharply reached 2,000 RPKM in S3 (Figure 4, Additional file 5). However, the *LbCCD4* expression obviously declined from S1 to S3 (Figure 4). These results suggest that more carotenoids are degraded in BF than in RF.

### Comparative analysis of the carotenogenesis-related gene expression in *L. barbarum* and *L. ruthenicum* fruits via qRT-PCR

**Figure 5:**
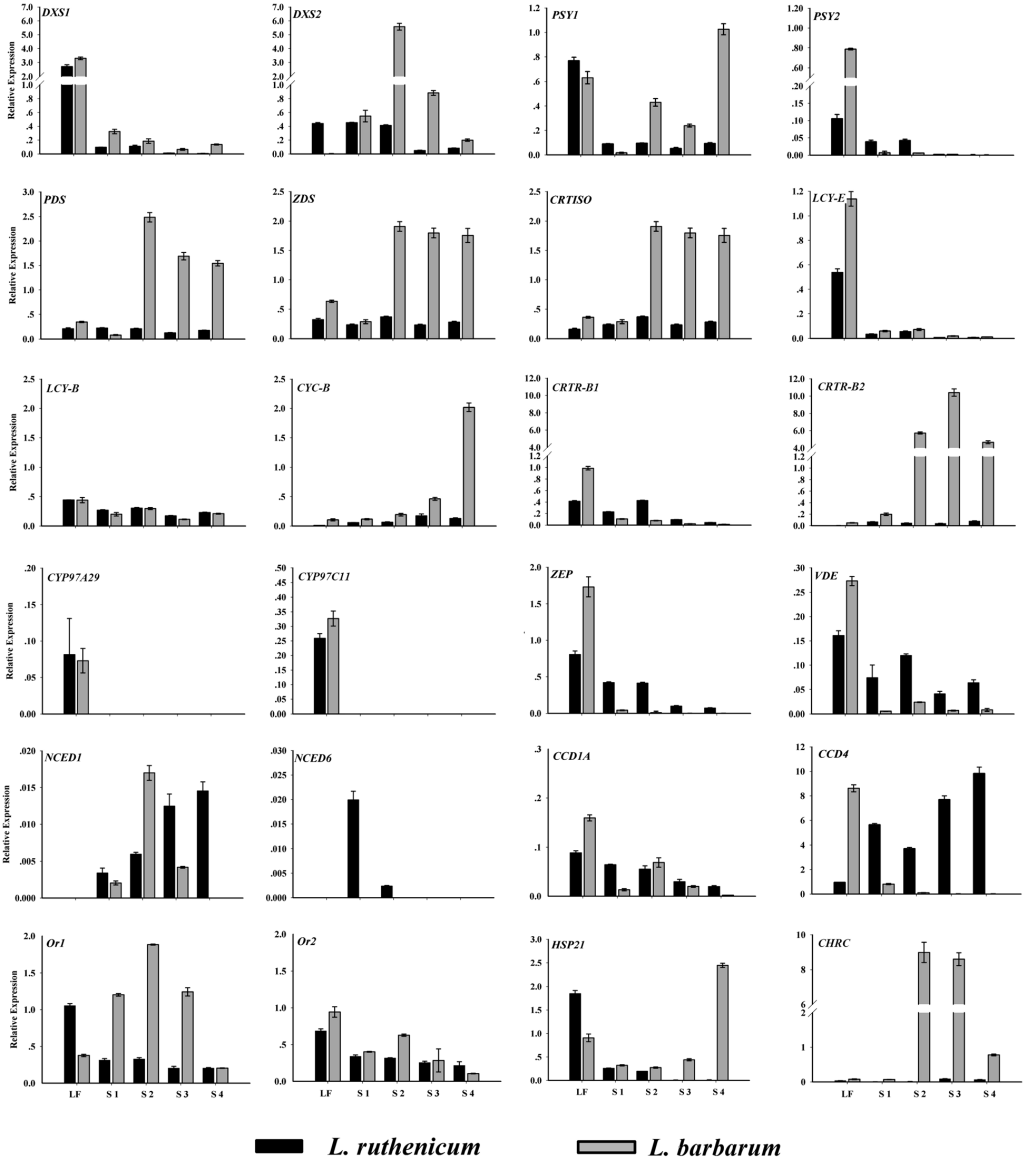
**Expression patterns of carotenoid-related genes in ripening RF (** ***L. barbarum*** **; gray bars) and BF (** ***L. ruthenicum*** **; black bars).** The expression of *Actin1* was used to normalize the mRNA levels for each sample. Three replicates were performed for each sample. LF, leaf samples; the fruit developmental stages (S1-S4) shown are identical to those depicted in Figure 1. *DXS, 1-deoxy-D-xylulose 5-phosphate-synthase; PSY, phytoene synthase; PDS, phytoene desaturase; Z-ISO, 15-cis-ζ-carotene isomerase; ZDS, ζ-carotene desaturase; CRTISO, carotene isomerase; LCY-B, lycopene β-cyclase; CYC-B, chromoplast-specific lycopene β-cyclase; CRTR-B, non-heme di-iron carotenoid β-ring hydroxylase; CYP97A29, P450 carotenoid β-ring hydroxylase; CYP97C11, P450 carotenoid ε-ring hydroxylase; NCED, 9-cis-epoxycarotenoid dioxygenase; CCD, carotenoid cleavage dioxygenase; CHRC, Chromoplast-specific carotenoid-associated protein; Or, orange; HSP21, heat shock protein 21; ZEP, zeaxanthin epoxidase; VDE, violaxanthin de-epoxidase*.

To confirm the expression patterns of all 25 of the carotenogenesis-related genes during RF and BF ripening (S1-S4), qRT-PCR was used (Figure 5). Consistent with the findings from the RNA-seq data, the *LrCHRC* transcripts were very low in BF, while the *LbCHRC* transcripts were abundant in RF, particularly in the S2 and S3 stages. Both *Or* genes displayed constant expression during BF ripening, while the *LbOr1* transcript level was much higher (5.9- to 9.2-fold) in RF S1-S3 than in S4. The transcript abundance of *HSP21* was increased by 7.6-fold from S1 to S4 of RF, while it decreased to undetectable levels in S3 and S4 of BF.

As the first enzyme in the MEP pathway, DXS was shown to be a regulatory enzyme in tomato fruit carotenogenesis [47]. In *Lycium*, *DXS1* showed similar expression profiles in the two species examined here, with much higher mRNA levels (10-fold) in the leaves than in the fruit. Specifically, the *LrDXS2* transcripts were equally abundant in the leaves during S1 and S2, before decreasing in S3 and S4, while the *LbDXS2* transcripts increased during the color-break stage (S2) by 10-fold and gradually decreased thereafter (Figure 5). The transcripts of the putative chromoplast-specific genes (*PSY1*, *CYC-B* and *CRTR-B2*) increased dramatically (by 64-, 17- and 53-fold, respectively) during RF ripening, whereas they were consistently expressed at relatively low levels throughout BF ripening (Figure 5). Similarly, the *PDS*, *ZDS*, and *CRTISO* transcript abundance was low during BF ripening but increased (by 30-, 6.7- and 6.3-fold, respectively) at the color-break stage (S2) in RF and remained high thereafter (Figure 5). The *Z-ISO* transcript was not detected in the leaves or fruits of either species via qRT-PCR. Similarly, the lutein synthesis genes *LCY-E*, *CYP97A29* and *CYP97C11* were expressed at low levels or were undetectable in the leaves and fruits of both species (Figure 5). The *ZEP* and *VDE* genes, which act downstream of zeaxanthin, showed similar expression profiles, with moderate transcript abundance in the leaves of both *L. barbarum* and *L. ruthenicum*, where they likely participate in the xanthophyll cycle. In the fruits however, both genes were consistently expressed during the ripening of BF but were barely detected in RF (Figure 5).

The transcript abundance of *NCED1* increased gradually throughout BF ripening, while in RF, it peaked at S2 and decreased thereafter (Figure 5). No *NCED6* transcripts were detected in the leaves or fruits of either species, except for extremely low levels in S1 and S2. In RF, the expression profile of *LbCCD1A* was similar to *LbNCED1*, while in BF, *LrCCD1A* was anti-correlated with *LbNCED1. LrCCD4* was highly expressed in BF, particularly in the late developmental stages, while *LbCCD4* was expressed at relatively low levels in RF and gradually decreased throughout the ripening process (Figure 5).

### Functional analysis of the key carotenoid biosynthesis enzymes from *L. barbarum* and *L. ruthenicum*

**Figure 6:**
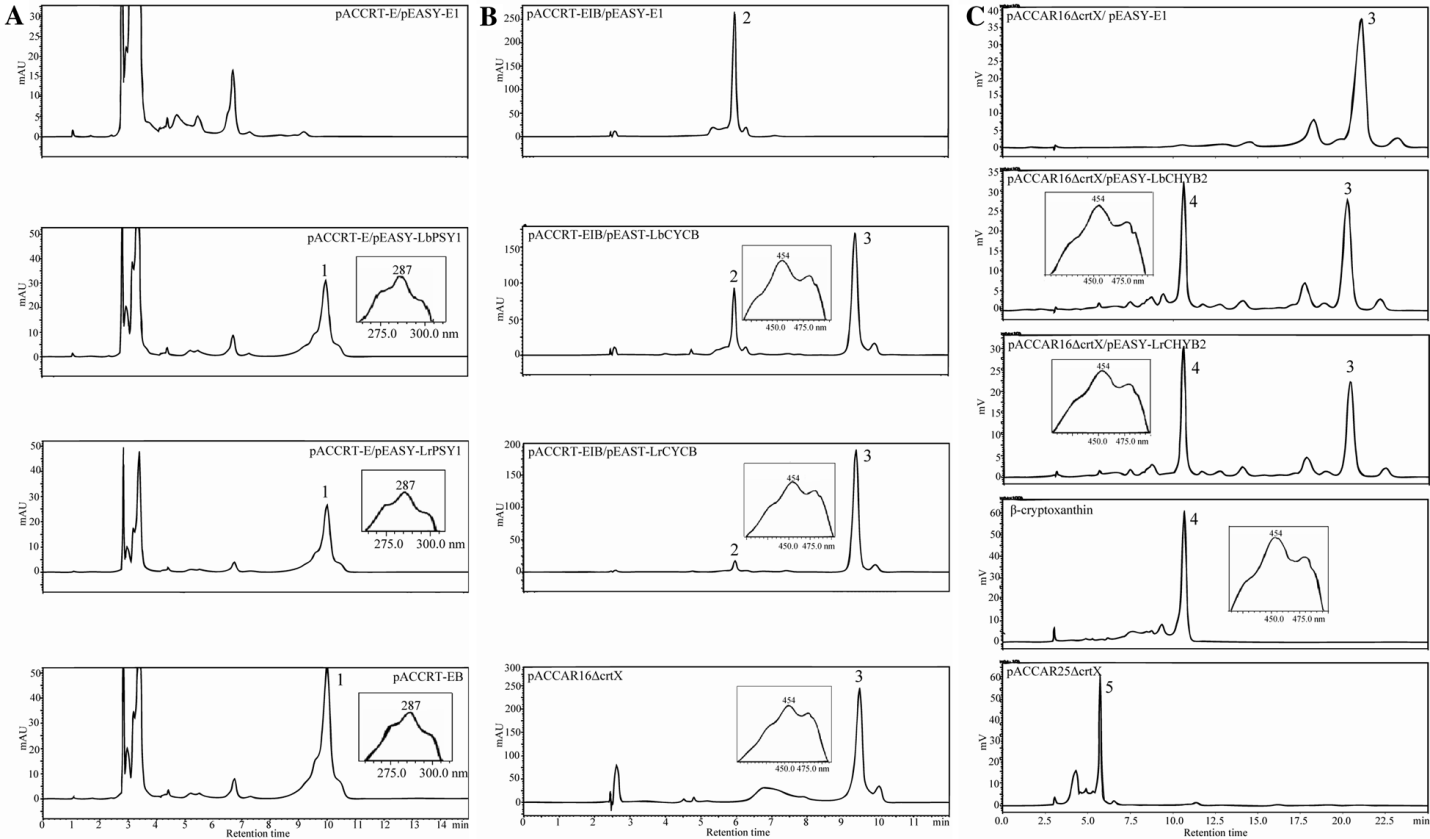
**Pigments produced in** ***E. coli*** **in the functional analysis for PSY1, CYC-B and CRTR-B2 of both species.** In the functional analysis of PSY1 **(A)**, pigments extracted from *E. coli* cells harboring pACCRT-E, the engineered plasmid producing GGPP, and pEASY-E1, the empty vector; plasmids pACCRT-E and pEASY-LbPSY1, which encodes LbPSY1; plasmids pACCRT-E and pEASY-LrPSY1, which encodes LrPSY1; and pACCRT-EB, the engineered plasmid producing phytoene (peak 1) as a positive control. The absorption spectra of phytoene are presented in the boxes with retention times of 10.0 min. In the functional analysis of CYC-B **(B)**, the plasmids are pACCRT-EIB, the engineered plasmid producing lycopene (peak 2), and pEASY-E1; pACCRT-EIB and pEASY-LbCYC-B, which encodes LbCYC-B; pACCRT-EIB and pEASY-LrCYC-B, which encodes LrCYC-B; and pACCAR16ΔcrtX, the engineered plasmid producing β-carotene (peak 3) as a positive control. The absorption spectra of β-carotene are presented in the boxes with retention times of 9.4 min. In the functional analysis of CRTR-B2 **(C)**, the plasmids are pACCAR16ΔcrtX (producing β-carotene, peak 3) and pEASY-E1; pACCAR16ΔcrtX and pEASY-LbCRTR-B2, which encodes LbCRTR-B2; pACCAR16ΔcrtX and pEASY-LrCRTR-B2, which encodes LrCRTR-B2; and pACCAR25ΔcrtX, the engineered plasmid producing zeaxanthin (peak 5). Beta-cryptoxanthin standard was used as the indicator of peak 4. The absorption spectra of β-cryptoxanthin are presented in the boxes with retention times of 10.5 min.

To verify the functionality of the key carotenoid biosynthesis enzymes identified in the two *Lycium* species, the bioactivities of PSY1, CYC-B and CRTR-B2 were tested in *E. coli*. Previous studies have demonstrated that these are rate-controlling enzymes in chromoplast-specific carotenoid biosynthesis [18,19,21]. The carotenoids in transformed *E. coli* cells were detected using high-performance liquid chromatography (HPLC) (Figure 6). The positive controls (Table 2) showed the expected absorbance spectra corresponding to phytoene, β-carotene and zeaxanthin. For the functional assays, the peaks of the carotenoids isolated from the bacteria containing the substrate synthesizing plasmids (pACCRT-E for GGPP, pACCRT-EIB for lycopene, pACCAR16ΔcrtX for β-carotene), coupled with the vectors containing the *Lycium* enzymes, represented the peaks for phytoene, β-carotene and β-cryptoxanthin. Beta-cryptoxanthin is an intermediate for zeaxanthin, and therefore is an indicator of insufficient CRTR-B2 hydroxylase activity *E. coli* (Figure 6) [48,49]. Overall, these results suggest similar PSY1, CYC-B and CRTR-B2 bioactivities between *L. barbarum* and *L. ruthenicum*.

**Table 2:** **Construct design for enzymatic assays in** ***E. coli***

**Genes to be analysed**	**Plasmids contained in** ***E. coli*** **(BL21) and the carotenoid** ^**a**^ **being produced (in parentheses)**		
	**Negative control**	**Functional assays**	**Positive control**
*LbPSY1/LrPSY1*	pACCRT-E + pEASY-E1	pACCRT-E + pEASY-*LbPSY1* or pACCRT-E + pEASY-*LrPSY1*	pACCRT-EB
	(GGPP)		(phytoene)
*LbCYC-B/LrCYC-B*	pACCRT-EIB + pEASY-E1	pACCRT-EIB + pEAST-*LbCYC-B* or pACCRT-EIB + pEASY-*LrCYC-B*	pACCAR16ΔcrtX
	(lycopene)		(β-carotene)
*LbCRTR-B2/LrCRTR-B2*	pACCAR16ΔcrtX + pEASY-E1	pACCAR16ΔcrtX + pEASY-*LbCRTR-B2* or pACCAR16ΔcrtX + pEASY-*LrCRTR-B2*	pACCAR25ΔcrtX
	(β-carotene)		(zeaxanthin)

^a^GGPP is an exception.

## Discussion

### The fruits of two valuable *Lycium* species show opposite carotenoid accumulation patterns

The red pigmentation of the ripe *L. barbarum* fruit is due to the high accumulation of specific carotenoids [41,46]. Unlike RF, the ripe fruit of *L. ruthenicum* is deep purple in color with a high petunidin content produced by the anthocyanin pathway [42]. To unravel the molecular regulatory basis for the differences in carotenoid accumulation between RF and BF, we first characterized the carotenoid compositional changes during fruit ripening in both species.

The phytochemical analysis revealed that the carotenoid accumulation increased during RF ripening (Additional file 1). As shown in Figure 2, the zeaxanthin precursor β-cryptoxanthin accumulated at consistently low levels during the RF ripening process, which was accompanied by a high level of zeaxanthin accumulation, consistent with previous studies (Additional files 1 and 2) [46]. These results suggest that the flux through the carotenoid pathway in RF is primarily directed into the β, β-carotene branch to produce zeaxanthin. At the same time, the chloroplastic carotenoids lutein and violaxanthin, present in pre-ripe S1 green RF, gradually decreased during fruit development (Additional file 2). Beta-carotene is also a chloroplastic carotenoid, and it declined from S1 to S2 in RF; however, as another intermediate of zeaxanthin biosynthesis, it increased from S2 to S4, consistent with the zeaxanthin accumulation (Additional file 1).

In BF, the products of both the ε, β-carotene and β, β-carotene branches of the carotenoid pathway, namely lutein and violaxanthin, gradually decreased to undetectable levels during ripening (Additional files 1 and 3). In contrast to the results for RF, the carotenoid compositions did not change during BF ripening, and the content of all of the existing compositions gradually declined to undetectable levels (Additional file 3). It is interesting to reveal the mechanisms underlying the different carotenoid accumulation patterns between RF and BF.

### The failure in chromoplast development results in no carotenoid accumulation in ripe BF

In fruits and flowers, a large abundance of carotenoids can be stored in the chromoplasts. Following the research on the *Or* gene, the formation of the chromoplast was recognized as a vital factor for carotenoid accumulation [50]. In the *Or* cauliflower mutant, the failure in chromoplast formation blocked the biosynthesis and accumulation of carotenoids [27]. In transgenic *Or*-overexpressing Arabidopsis and rice, the chromoplast differentiation occurred in the calli of both species and induced the biosynthesis of the carotenoids [28,51]. In addition, *CHRC* and *HSP21* were also shown to play significant roles in chromoplast development and carotenoid storage [52,53].

In this study, we observed that orange, globular chromoplasts existed in the cells of the ripe RF (Figure 3B), consistent with the abundant carotenoid accumulation. Likewise, consistent with the poor accumulation of carotenoids in BF, these organelles were not observed in the ripe BF (Figure 3D). Therefore, the development of the chromoplasts may be the primary cause of the differences in carotenoid accumulation between RF and BF. The expression profiles of the chromoplast-related genes (*Or*, *CHRC* and *HSP21*) during RF and BF development also supported this speculation. *Or1*, *CHRC* and *HSP21* all showed much higher expression levels in RF compared to BF (Figures 4 and 5).

Within the chromoplast, the carotenoids and the CHRC protein are predominantly stored in lipoprotein fibrils [54]. Distinct from the plastoglobules in the chloroplasts, these fibrils are characterized by a high homogeneity of apolar compounds, most of which are esterified xanthophylls [55]. In potato tubers, a positive correlation between the total carotenoid content and the esterified xanthophyll fraction was observed, suggesting that esterification facilitates the accumulation of these lipophilic compounds within the plastids [56]. Recently, this viewpoint was also verified in apples (*Malus x domestica* Borkh) [57]. The majority of the zeaxanthin that accumulates in the ripe, red fruits of *L. barbarum* is esterified to zeaxanthin-dipalmitate [46]. Given the very high content of zeaxanthin in RF determined here, it is possible that, similar to potatoes and apples, the esterification of zeaxanthin may be a key regulatory step in carotenoid accumulation in *L. barbarum* fruit.

### The species differences in the carotenoid accumulation are not due to differences in the functions of key enzymes

In addition to the chromoplast development, another explanation for the large species-specific differences in the total carotenoid content of *Lycium* fruits could be altered by the functionality of the carotenoid biosynthetic enzymes. Given that chloroplastic carotenoids are indispensable for plant survival and to investigate this possibility, we focused on key enzymes that may be not necessary for chloroplastic carotenoid biosynthesis. These are the chromoplast-specific PSY1, CYC-B and CRTR-B2 enzymes. Protein sequence comparisons revealed up to 97% identity between species, with no insertion/deletion or frame shift mutations indicative of non-functional proteins in *L. ruthenicum* (Table 1). Furthermore, the protein expression in *E. coli* revealed that all of the enzymes from both species were equally functional in catalyzing their respective carotenoid substrates (Figure 6). Therefore, the low carotenoid content in the BF of *L. ruthenicum* is unlikely to be due to reduced activities of the carotenoid biosynthetic enzymes.

### The biosynthesis of zeaxanthin in RF is regulated at the transcriptional level

Increasing evidence suggests that the carotenoid content in the chromoplasts is predominantly regulated at the transcriptional level [16]. The ripening of the tomato fruit is one of the best studied systems for the regulation of carotenoid biosynthesis and accumulation in the chromoplasts. Changes in the production of carotenoids associated with tomato fruit ripening are mainly controlled via the transcriptional regulation of biosynthetic genes [58]. During RF, the zeaxanthin accumulation was significantly correlated with the upregulated expression of the upstream biosynthetic genes *DXS2*, *PSY1*, *PDS*, *ZDS*, *CRTISO*, *CYC-B*, and *CRTR-B2* and the very low expression of the downstream gene *ZEP* (Figures 4 and 5; Additional file 5). In tomatoes, *DXS1* is ubiquitously expressed and shows the highest expression levels during fruit ripening, while the *DXS2* transcripts are not detected in the fruit [59]. Here, the *DXS1* transcript was detected at very low levels in RF, whereas *DXS2* was expressed more highly, especially in S2 (Figure 5). This difference between tomatoes and *L. barbarum* may indicate a functional divergence of paralogous genes in the different species [60]. During RF development, the upregulation of the chromoplast-specific genes *LbPSY1*, *LbCYC-B* and *LbCRTR-B2* indicated the presence of chromoplast-specific carotenoid biosynthesis in RF (Figures 4 and 5). The *PDS*, *ZDS* and *CRTISO* genes showed similar expression patterns during RF ripening, with a sharp increase from S1 to S2 that was maintained until S4 (Figure 5). Therefore, it is possible that the transcription of these three genes is controlled by the same mechanism or regulatory factor(s). Due to the failure in chromoplast formation during BF development, all of the abovementioned genes were expressed with generally lower levels in BF than those in RF (Figure 5; Additional file 5).

LCY-E and two P450 family hydroxylases (CYP97A29 and CYP97C11) are primarily involved in the biosynthesis of lutein [61]. The qRT-PCR results showed that these three genes exhibit a similar expression pattern in both *Lycium* species, being highly expressed in the leaves and expressed at low levels in the fruit (Figure 5). This was consistent with the low or no lutein accumulation in RF and BF.

### Carotenoid degradation may continuously occur in the BF of *L. ruthenicum*

The relatively low transcript levels (but not no transcript) of the carotenoid biosynthetic genes in all four BF stages (Figure 5, Additional file 5) suggested that although the chromoplasts were not well formed in BF, the biosynthesis of the carotenoids still occurred to a small extent. However, the content of the carotenoids decreased to undetectable levels during BF ripening (Additional file 2). Therefore, we speculated that the carotenoid degradation occurred during the development of BF. In plants, the degradation of carotenoids is catalyzed by a family of CCDs, which contribute to the overall control of the cellular carotenoid content [6,22,62]. Arabidopsis has nine CCD family members, five of which have been classified as ABA-related AtNCEDs, and the remaining are AtCCD1, AtCCD4, AtCCD7 and AtCCD8 [63]. Of these, CCD4 has been proven to play a decisive role in the regulation of the carotenoid content in some plant organs [64], including chrysanthemum petals [23], peach fruits [24,25] and potato tubers [26]. Interestingly, our results confirmed that *LrCCD4* was highly expressed during BF development (Figures 4 and 5; Additional file 5). These results suggest that the low activity biosynthetic carotenoids were gradually degraded by the highly active LrCCD4 during BF ripening, further resulting in almost undetectable carotenoid levels in the ripe BF. In contrast, the transcripts of *LbCCD4* showed a decreasing trend during RF ripening, and its expression level was much lower in RF than in BF (Figure 5, Additional file 5). Therefore, the lower rate of carotenoid degradation may be another factor for the increased carotenoid accumulation in RF compared to BF.

## Conclusions

In conclusion, a regulatory model for the species-specific differences in carotenoid accumulation in *L. barbarum* and *L. ruthenicum* fruits has been proposed. The development of carotenoid sink organelles (chromoplasts) is likely the primary cause of the differences in carotenoid accumulation between RF and BF. In RF, based on the formation of chromoplasts, a high flux towards zeaxanthin, which is regulated at the transcriptional level, combined with a low rate of carotenoid degradation concurrently determine the observed accumulation of high levels of zeaxanthin. In BF, where the chromoplasts are not formed, small amounts of carotenoids are biosynthesized, but they are mostly degraded by LrCCD4; therefore, no carotenoids can be detected in ripe BF. The esterification of zeaxanthin in RF may be a possible regulatory step for carotenoid biosynthesis, which still requires further investigation. This study has improved our understanding of the regulatory mechanisms controlling the levels of important medicinal and nutritional compounds in *Lycium*.

## Methods

### Plant material

The *L. barbarum* and *L. ruthenicum* samples (mature leaves and fruits at four developmental stages) used in this study were collected from Zhongning County, the Ningxia Hui Autonomous Region and the Turpan Desert Botanical Garden of the Chinese Academy of Sciences, China. The samples of the four fruit developmental stages (for both RF and BF) were harvested based on the phenotype of the fruit epidermis (Figure 1): the green fruit stage (S1, 3 days before color break), the color-break stage (S2), the light-color stage (S3, 3 days after break) and the ripe fruit stage (S4, 6 days after break). For each developmental stage, more than twenty fruits were collected randomly and were then separated into three replicate groups. After harvest, each group of fruits was weighed, frozen in liquid nitrogen, and stored at −80°C until further use.

### Carotenoid extraction

The carotenoids were extracted from the fruits as previously described [56]. Briefly, the fruits were ground into a fine powder with liquid nitrogen and extracted three times using 5 ml of hexane/acetone/ethanol (2:1:1, v/v/v; with 0.1% butylated hydroxytoluene) via an ultrasonic treatment for 30 min until the sample was colorless. After centrifugation (4000 × *g* for 10 min at 4°C), the extracts were combined into a 50-ml tube, followed by shaking with 5 ml of NaCl-saturated solution for 1 min, and the supernatant was collected. The residue was partitioned with 5 ml of hexane and repeated three times, and all of the supernatants were combined and dried in a Vacufuge Plus vacuum concentrator (Eppendorf, Germany). Dichloromethane (2 ml) was added for the HPLC analysis of the samples that did not require saponification. For the samples that required saponification, the residue was dissolved in 2 ml of methyltert-butylether (MTBE), after which, 2 ml of a 15% (w/v) KOH/methanol solution was added for the saponification for 6 h in the dark under nitrogen [46]. After the saponification, the solutions were partitioned with 2 ml of MTBE and 4 ml of NaCl-saturated solution, and the supernatant was collected. The lower aqueous layer was repeatedly partitioned three times with 2 ml of MTBE. The supernatants were pooled, vacuum dried, and dissolved in 2 ml of dichloromethane for the HPLC analysis and the total carotenoid quantification.

### HPLC analysis

The HPLC analysis was carried out on an LC-20A liquid chromatograph (Shimadzu, Japan) with two LC-20AT pumps and an SPD-M20A UV/VIS detector. All of the separations were performed using a reverse-phase C_30_ carotenoid column (250 × 4.6-mm i.d., 5-μm particle size, YMC, Japan) coupled to a 23 × 4.0-mm guard column. The data were acquired and processed using Shimadzu LC solution software. A binary mobile phase of methanol/acetonitrile (3:8, v/v) (A) and dichloromethane/hexane (1:1, v/v) (B) was used with the following gradient elution: 95% A and 5% B initially, decreased to 50% A in 15 min and returned to 95% A in 20 min, then maintained until 25 min. The column temperature was maintained at 30°C, with a flow rate of 1 ml/min and a detection wavelength of 450 nm. The quantification was performed using a calibration curve generated with commercially available β-carotene, β-cryptoxanthin and zeaxanthin standards (Sigma-Aldrich) (Additional file 6). For the preparation of the standard curves, a mixture of zeaxanthin, β-cryptoxanthin and β-carotene was dissolved into five concentrations (zeaxanthin: 2.5, 5, 25, 50 and 100 μg ml^−1^; β-cryptoxanthin: 0.5, 1, 5, 10 and 20 μg ml^−1^; β-carotene: 0.5, 1, 5, 10 and 20 μg ml^−1^). Three standard curves were each prepared by plotting the concentration of the carotenoid standard to its area. The regression equations and correlation coefficients (*R*^2^) of the standard curves are shown in Additional file 7. Lutein and violaxanthin were identified via their absorption spectra and based on previous reports. All of the spectra of the identified carotenoids are listed in Additional file 8. The total carotenoid content was estimated using a spectrophotometer using zeaxanthin for the standard curve drawing.

### Light microscopy

The tissues were treated for microscopy as previously described [36]. Briefly, immediately after excision with a sterile razor blade, the young fruits (S1) and ripe fruits (S4) of *L. barbarum* and *L. ruthenicum* (mesocarp only, 1-mm^2^ sections) were fixed in 3.5% glutaraldehyde solution for 1 h in darkness. The young fruit tissue was disrupted at 65°C in a solution of disodium EDTA (EDTA-Na_2_; 0.1 M, pH 9.0) for 20 min, followed by maceration with clean forceps on glass microscope slides. The ripe-fruit mesocarp was disrupted in a solution of EDTA-Na_2_ (0.1 M, pH 9.0) at room temperature. The samples were imaged using a ZEISS Axioplan2 imaging microscope (Carl Zeiss, Germany). The images were captured with a ZEISS Axiocam MRC digital camera and Axiovision 4.6 software.

### Gene cloning and sequence analysis

Total RNA was isolated from the *L. barbarum* and *L. ruthenicum* tissues using TRIzol Reagent (Invitrogen, USA). For the gene cloning for each species, the five RNA samples (from the leaf and four developmental fruits) were combined and 20 ng of the RNA was reverse transcribed into cDNA using SuperScript II™ (Invitrogen, USA) according to the manufacturer’s instructions. A unigene library of *L. barbarum* (unpublished data) was searched using the carotenogenesis-related genes of tomatoes, and 25 putative carotenogenesis-related unigenes from *L. barbarum* were identified, namely *DXS1*, *DXS2*, *PSY1*, *PSY2*, *PDS*, *Z-ISO*, *ZDS*, *CRTISO*, *LCY-B*, *CYC-B*, *LCY-E*, *CYP97A29*, *CYP97C11*, *CRTR-B1*, *CRTR-B2*, *ZEP*, *VDE*, *NCED1*, *NCED6*, *CCD1A*, *CCD4*, *CHRC*, *Or1*, *Or2* and *HSP21* (Table 1). The primers (Additional file 9) were designed to amplify all of the genes containing a complete ORF in *L. barbarum* and *L. ruthenicum*. The PCR was performed in 25-μl mixtures containing 1 U of PrimeSTAR HS DNA Polymerase (Takara, China), 5 μl of PrimeSTAR buffer (5×), 0.2 mM of each dNTP, 0.3 μM of each primer and 50 ng of cDNA. The following conditions were used: denaturation at 98°C for 5 min; 30 cycles of 98°C for 10 s, 60°C for 15 s, 72°C for 2 min, and a final 10 min extension at 72°C. The products were gel purified, ligated into the pMD19-T vector (Takara, China) and verified via sequencing (Sangon, China). The predicted coding and amino acid sequences for each gene were reciprocally compared between *L. barbarum*, *L. ruthenicum* and *S. lycopersicum* using DNAMAN (Table 1). The sub-cellular localization of the protein sequences was predicted using ProtComp (http://www.softberry.com/berry.phtml) (Table 1).

### RNA-seq expression profiling of the carotenogenesis-related genes during the fruit ripening process (S1-S3) of RF and BF

The RNA-seq data of two replicates for the three developmental stages (S1-S3) of RF and BF were used to characterize the expression profiles of the carotenogenesis-related genes. The gene expression levels were quantified in RPKM. Twenty-five carotenogenesis-related genes were analyzed, including two genes from the MEP pathway: *DXS1* and *DXS2*; fifteen carotenoid biosynthetic genes: *PSY1*, *PSY2*, *PDS*, *Z-ISO*, *ZDS*, *CRTISO*, *LCY-B*, *CYC-B*, *LCY-E*, *CRTR-B1*, *CRTR-B2*, *CYP97A29*, *CYP97C11*, *ZEP* and *VDE*; four carotenoid cleavage genes: *NCED1*, *NCED6*, *CCD1A* and *CCD4*; and four chromoplast-related genes: *CHRC*, *Or1*, *Or2* and *HSP21* (Additional file 5).

### Functional analyses of *PSY1*, *CYC-B* and *CRTR-B2* in *E. coli*

To test the differences between the functions of the key genes in *L. barbarum* and *L. ruthenicum*, three rate-controlling carotenoid biosynthesis genes from each species (*LbPSY1*/*LrPSY1*, *LbCYC-B*/*LrCYC-B* and *LbCRTR-B2*/*LrCRTR-B2*) were cloned into pEASY-E1 (Trans, China). The carotenoid producing plasmids were kindly provided by Dr. Norihiko Misawa. The positive controls, negative controls and gene functional assays are described in Table 2. The plasmids were transformed into *E. coli* BL21 (DE3) and grown overnight at 37°C on Luria-Bertani (LB) solid medium with appropriate antibiotics (30 μg/ml chloramphenicol for the positive controls containing only one plasmid or 100 μg/ml ampicillin and 30 μg/ml chloramphenicol for selecting two plasmids). The selected positive colonies were shaken overnight at 37°C in LB liquid medium with the appropriate antibiotics and then isopropyl β-D-1-thiogalactopyranoside (IPTG) was added to a concentration of 1.0 mM. The bacterial cultures were incubated at room temperature in darkness for 24 h for the gene expression. The cells were harvested by centrifugation (3000 × *g* for 15 min at 4°C), washed in sterile distilled water, lyophilized and used for the carotenoid extraction and HPLC analysis as described above.

### Quantitative reverse transcription-PCR (qRT-PCR)

qRT-PCR was performed in five samples (the leaf and four developmental fruits) for each species. The cDNA was synthesized from 20 ng of RNA using a PrimeScript™ RT reagent Kit with gDNA Eraser (Perfect Real Time) (Takara, China). The qRT-PCR primers were designed using PRIMER3 software (Rozen and Skaletsky, 2000 [65]; listed in Additional file 9). The primers were designed to amplify the conserved sequences between *L. barbarum* and *L. ruthenicum.* The PCR was performed on a LightCycler^@^ 480 (Roche, USA) using SYBR® Premix Ex Taq™ (Perfect Real Time) (Takara, China). The reaction mixture contained 10.0 μl of 2 × SYBR® *Premix Ex Taq*™II, 2 μl of the cDNA solution (40 ng/μl), 0.8 μl of each primer (10 μM) and 6.4 μl of ddH_2_O, in a final volume of 20 μl. The amplification conditions were as follows: 95°C for 30 s, 40 cycles of 95°C for 5 s, 50°C for 30 s, and 72°C for 30 s. The relative expression levels were calculated using the ΔΔC_T_ method as described by Lyi *et al.* (2007) [66]. All of the data were normalized to *Lycium Actin1*.

### Accession numbers

The sequence data reported in this article can be found in GenBank under the following accession numbers: KC190187 and KC190188 for *LbPDS* and *LrPDS*, respectively; KF957678-KF957701 for *DXS1*, *DXS2*, *PSY1*, *PSY2*, *Z-ISO*, *ZDS*, *CRTISO*, *LCY-B*, *CYC-B*, *LCY-E*, *CRTR-B1*, *CRTR-B2*, *CYP97A29*, *CYP97C11*, *ZEP*, *VDE*, *NCED1*, *NCED6*, *CCD1A*, *CCD4*, *CHRC*, *Or1*, *Or2* and *HSP21* isolated from *L. barbarum*; KF957702-KF957725 for these 24 genes isolated from *L. ruthenicum*.

## Additional files

Additional file 1:
**Carotenoid concentrations (μg g** ^**−1**^ **fresh weight) in all four developmental stages (S1-S4) of** ***L. barbarum*** **and** ***L. ruthenicum*** **fruits.**
Additional file 2:
**Accumulation profiles of carotenoids in ripening RF revealed by HPLC.** Peak identification: (1) violaxanthin, (2) lutein, (3) β-carotene, (4) β-cryptoxanthin, (5) zeaxanthin. Additional file 3:
**Accumulation profiles of carotenoids in ripening BF revealed by HPLC.** Peak identification: (1) violaxanthin, (2) lutein, (3) β-carotene, (6) unidentified, (7) unidentified. Additional file 4:
**Phylogenetic trees for the carotenogenesis-ralated proteins from** ***L. barbarum*** **(Lb),** ***L. ruthenicum*** **(Lr) and other organisms.** The trees were constructed by MEGA 5.1 program [67] using the neighbor-joining method [68].The proteins in *Solanum lycopersicum* (Sl), *Capsicum annuum* (Ca), *Arabidopsis thaliana* (At), *Nicotiana tabacum* (Nt) and *Brassica oleracea* (Bo) include SlDXS1 (FN424051), SlDXS2 (FN424052), SlPSY1 (ABU40772), SlPSY2 (ABU40771), SlPDS (AGO05926), SlZ-ISO (XP_004252966), SlZDS (AGO05927), SlCRTISO (AAL91366), SlLCY-B (NP_001234226), SlCYC-B (AAG21133)*,*SlLCY-E (Y14387), SlCRTR-B1 (CAB55625), SlCRTR-B2 (CAB55626)*,* SlCYP97A29 (ACJ25969), SlCYP97C11 (ACJ25968), SlZEP (P93236), SlVDE (ACM92036), SlNCED1 (CAB10168), SlNCED6 (XP_004240215), SlCCD1A (AAT68187), SlCCD1B (AAT68188), SlCCD4 (XP_004246004), SlCHRC (ABC42191), SlHSP21 *(*AAB07023*);* CaTKT2 (CAA75778), CaPSY (P37272), CaPDS (P80093), CaZDS (Q9SMJ3), CaLCY-B (ADH04271), CaCCS *(*CAA54495*),* CaCRTR-B1 (CAA70888), CaCRTR-B2 (CAA70427), CaZEP (Q96375), CaFBN *(*CAA50750*);* AtCLA1 (AAC49368), AtPSY (AAM62787), AtPDS (AAL15300), AtZ-ISO (NP_001117264), AtZDS (AAM63349), AtCRTISO (NP_172167), AtLCY-B (AAB53337), AtLCY-E (AAB53336), AtCRTR-B2 *(*NP_200070), AtCRTR-B1 *(*AAC49443), AtCYP97A3 (AAL08302), AtCYP97C1 (AAM13903), AtZEP (BAB08942), AtVDE *(*AAC50032*),* AtNCED2 (AT4G18350), AtNCED3 (AT3G14440), AtNCED5 (AT1G30100), AtNCED6 (AT3G24220), AtNCED9 (AT1G78390), AtCCD1 (AT3G63520), AtCCD4 (AT4G19170), AtCCD7 (AT2G44990), AtCCD8 (AT4G32810), AtFBN (NP_192311), AtHSP21 (NP_194497); NtOr (AEV23056) and BoOr (ABH07405). Additional file 5:
**RNA-seq expression profiles of twenty-five carotenogenesis-related genes during fruit ripening (S1-S3) of RF and BF, and the expression profiles of orthologous genes in tomato.**
Additional file 6:
**Chromatogram of standard mixture.**
Additional file 7:
**Standard curves of zeaxanthin, β-cryptoxanthin and β-carotene.**
Additional file 8:
**Spectra of the carotenoids discussed in the sutdy.** (1) violaxanthin, (2) lutein, (3) β-carotene, (4) β-cryptoxanthin, (5) zeaxanthin, (6) unidentified, (7) unidentified. Additional file 9:
**Specific primers used for gene cloning and qRT-PCR.**
