# Supplementary material for: Comparative analysis of carotenoid accumulation in two goji (Lycium barbarum L. and L. ruthenicum Murr.) fruits
Source: BMC Plant Biol. 2014 Dec 16;14:269. doi: 10.1186/s12870-014-0269-4 (PMC4276078; doi:10.1186/s12870-014-0269-4)
Supplement: Additional file 1: — Carotenoid concentrations (μg g −1 fresh weight) in all four developmental stages (S1-S4) of L. barbarum and L. ruthenicum fruits. [file 12870_2014_269_MOESM1_ESM.pdf]

Additional file 1 - Carotenoid concentrations (μg g^{-1} fresh weight) in all four developmental stages (S1-S4) of *L. barbarum* and *L. ruthenicum* fruits.

Compound	S1	S2	S3	S4
L. barbarum				
Zeaxanthin	ND	ND	77.16 ± 28.26	381.60 ± 26.78
β-Cryptoxanthin	ND	ND	3.00 ± 0.05	17.59 ± 4.81
β-Carotene	12.91 ± 2.29	10.18 ± 2.76	16.70 ± 3.45	28.99 ± 6.46
Total carotenoids[a]	44.36 ± 2.38	21.70 ± 3.23	105.36 ± 28.26	508.90 ± 20.31
L. ruthenicum				
Zeaxanthin	ND	ND	ND	ND
β-Cryptoxanthin	ND	ND	ND	ND
β-Carotene	18.01 ± 1.04	8.48 ± 0.65	3.22 ± 0.44	ND
Total carotenoids[a]	34.46 ± 2.18	25.97 ± 2.00	9.57 ± 2.00	0.01 ± 0.00

[a]Only three major carotenoids (zeaxanthin, β-cryptoxanthin and β-carotene) were quantified for the HPLC results, while the total amount of carotenoids was measured by spectrophotometer method.
