# Supplementary figures and images for: Comparative analysis of carotenoid accumulation in two goji (Lycium barbarum L. and L. ruthenicum Murr.) fruits
Source: BMC Plant Biol. 2014 Dec 16;14:269. doi: 10.1186/s12870-014-0269-4 (PMC4276078; doi:10.1186/s12870-014-0269-4)

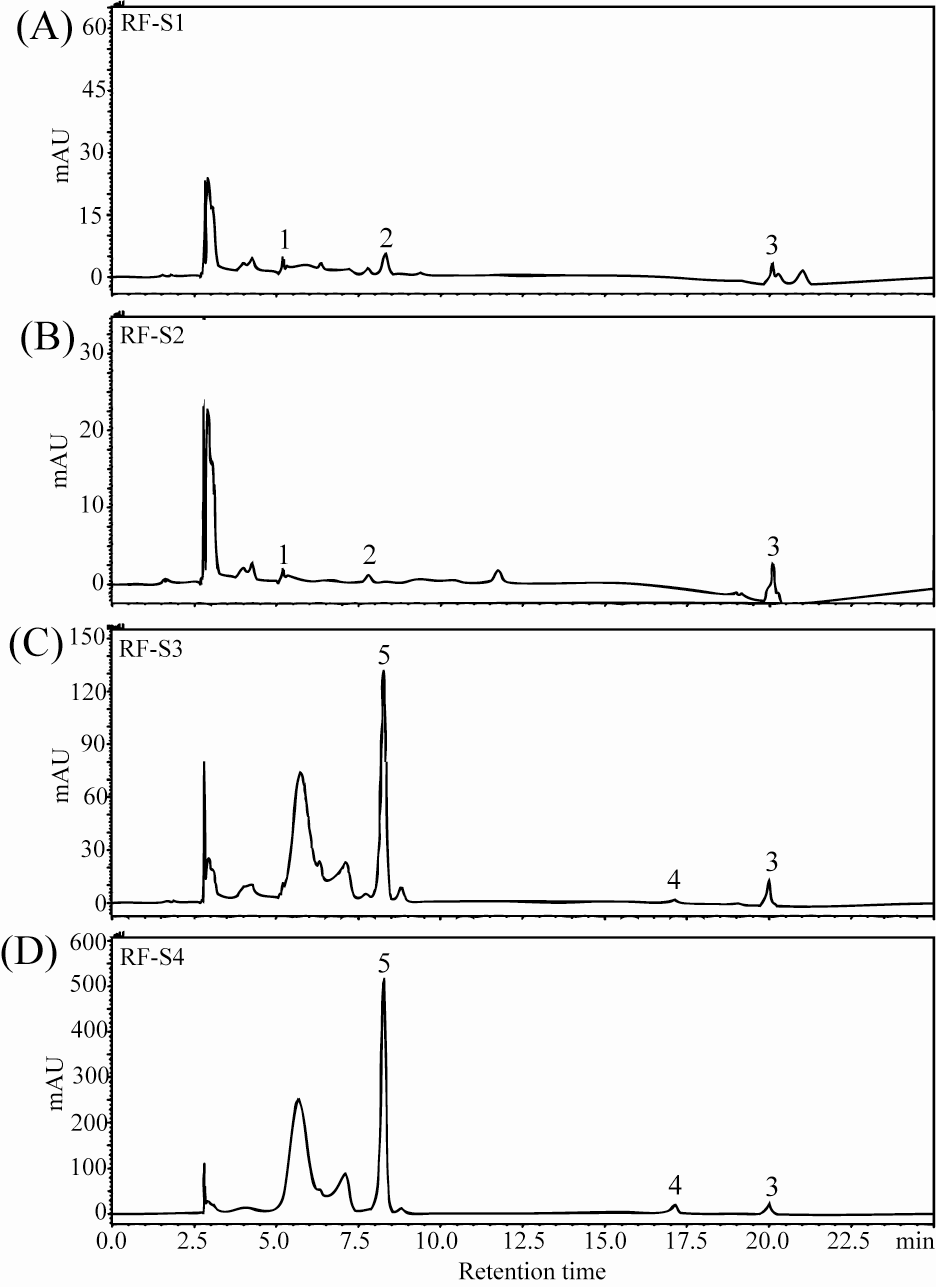

Supplement: Additional file 2: — Accumulation profiles of carotenoids in ripening RF revealed by HPLC. Peak identification: (1) violaxanthin, (2) lutein, (3) β-carotene, (4) β-cryptoxanthin, (5) zeaxanthin. [file 12870_2014_269_MOESM2_ESM.png]

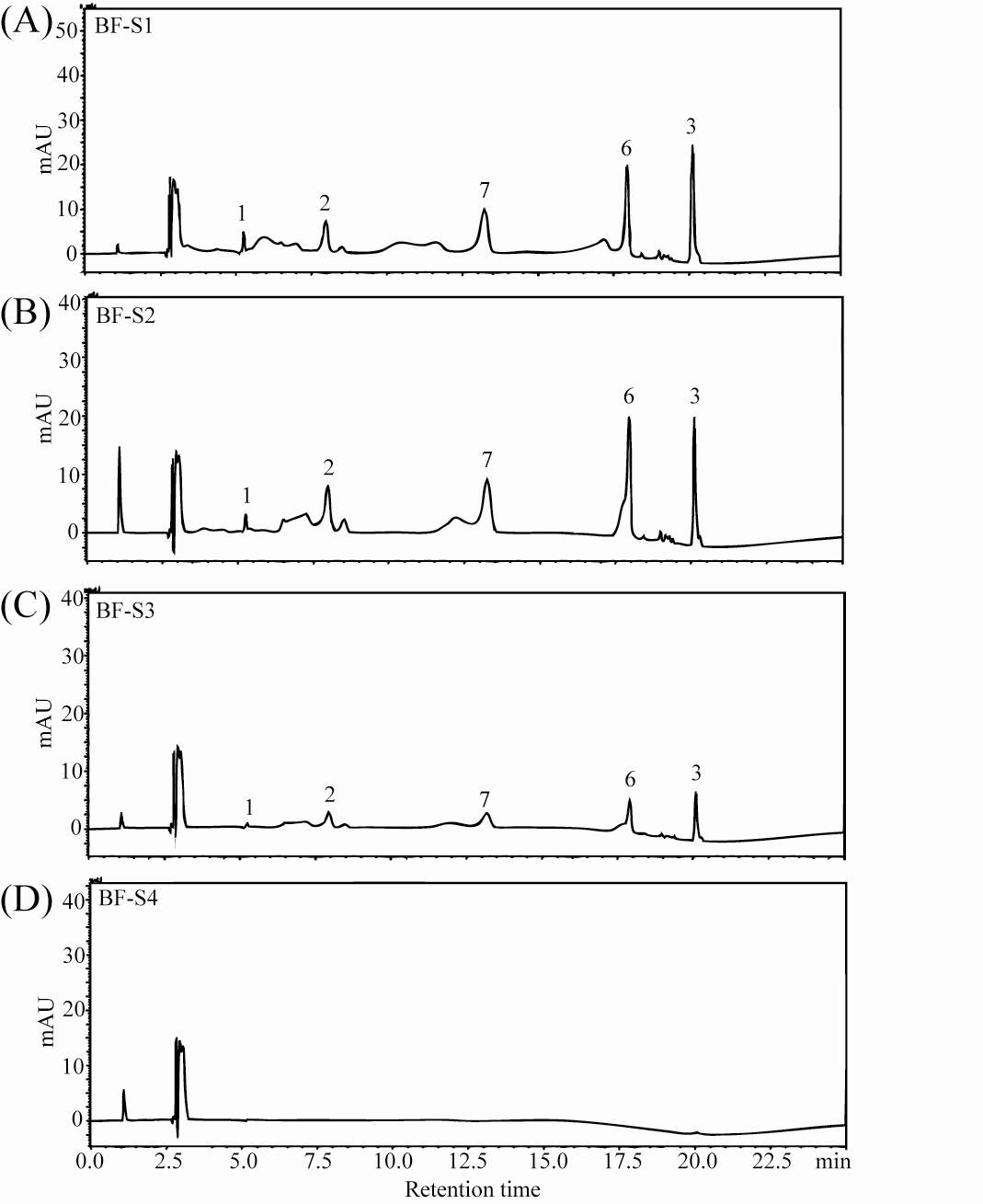

Supplement: Additional file 3: — Accumulation profiles of carotenoids in ripening BF revealed by HPLC. Peak identification: (1) violaxanthin, (2) lutein, (3) β-carotene, (6) unidentified, (7) unidentified. [file 12870_2014_269_MOESM3_ESM.png]

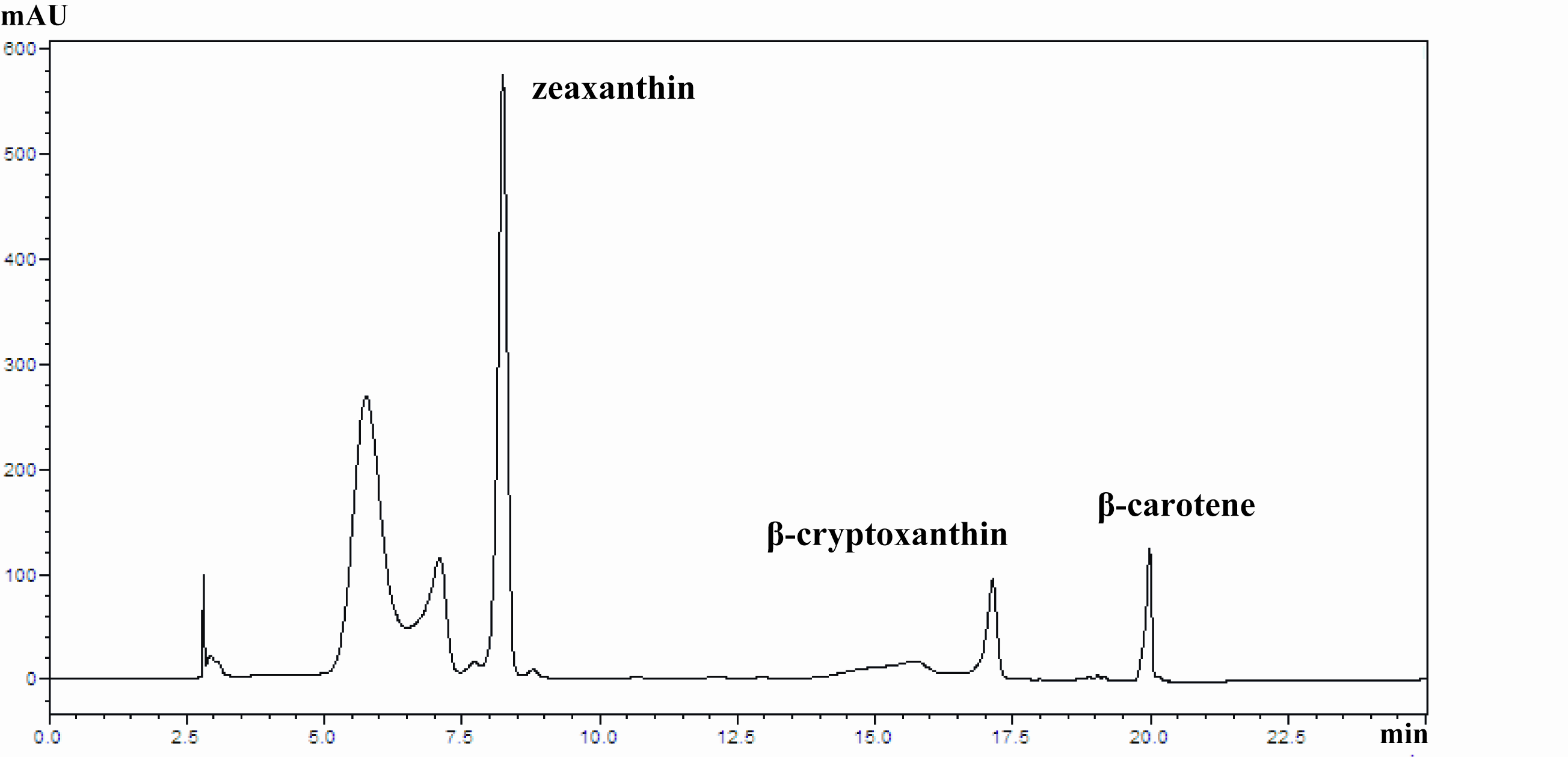

Supplement: Additional file 6: — Chromatogram of standard mixture. [file 12870_2014_269_MOESM6_ESM.png]

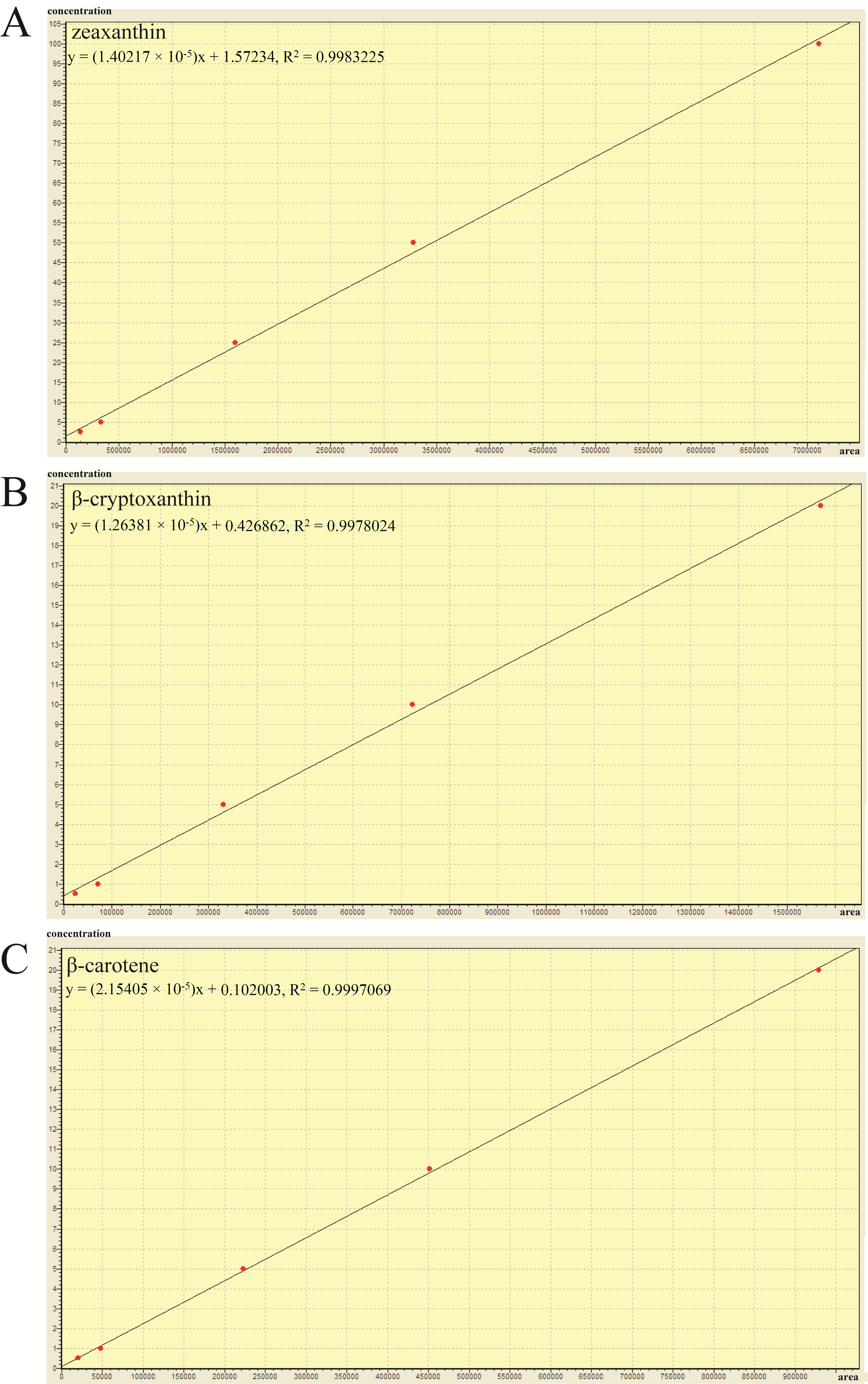

Supplement: Additional file 7: — Standard curves of zeaxanthin, β-cryptoxanthin and β-carotene. [file 12870_2014_269_MOESM7_ESM.png]

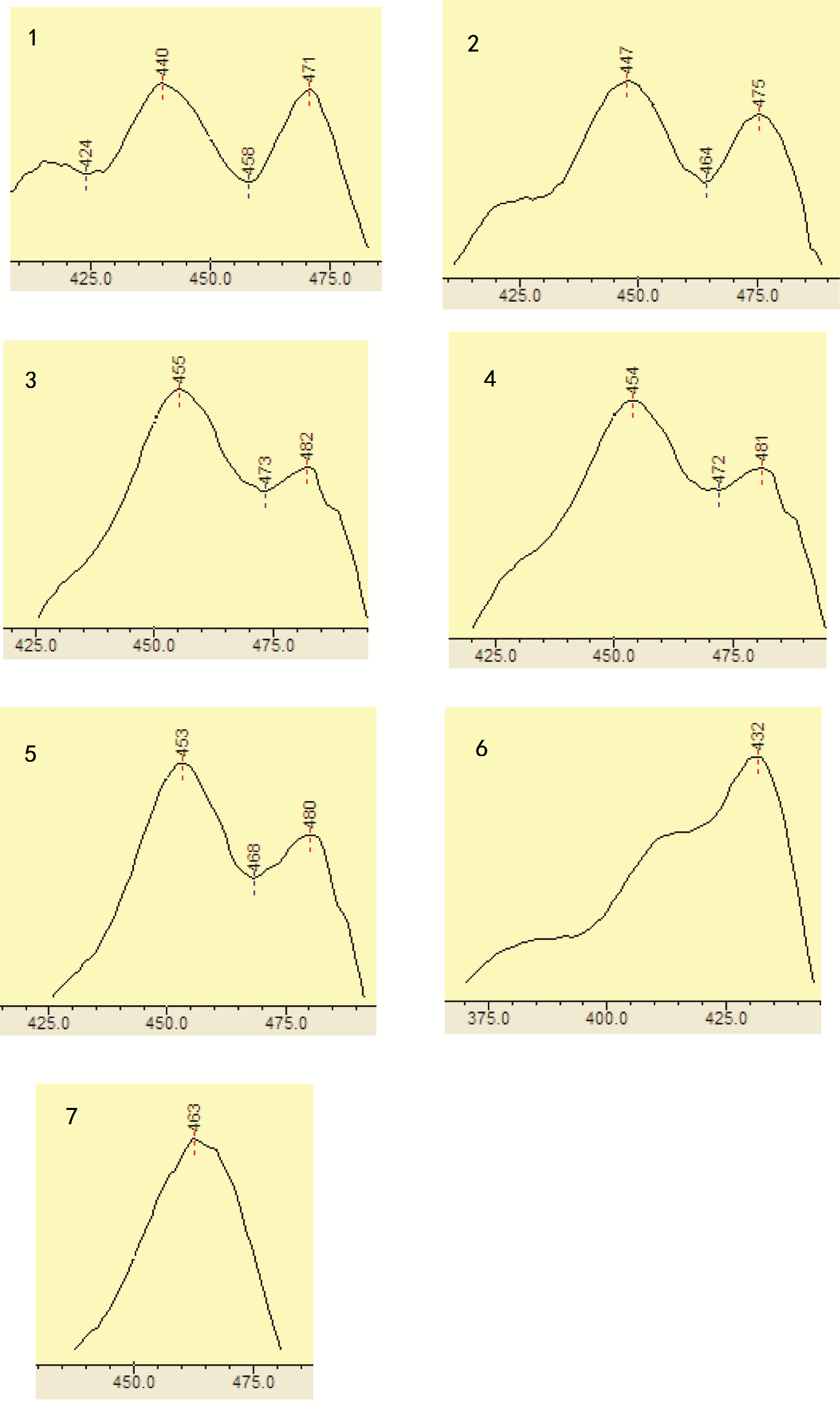

Supplement: Additional file 8: — Spectra of the carotenoids discussed in the sutdy. (1) violaxanthin, (2) lutein, (3) β-carotene, (4) β-cryptoxanthin, (5) zeaxanthin, (6) unidentified, (7) unidentified. [file 12870_2014_269_MOESM8_ESM.png]
