# Supplementary material for: Comparative analysis of carotenoid accumulation in two goji (Lycium barbarum L. and L. ruthenicum Murr.) fruits
Source: BMC Plant Biol. 2014 Dec 16;14:269. doi: 10.1186/s12870-014-0269-4 (PMC4276078; doi:10.1186/s12870-014-0269-4)
Supplement: Additional file 4: — Phylogenetic trees for the carotenogenesis-ralated proteins from L. barbarum (Lb), L. ruthenicum (Lr) and other organisms. The trees were constructed by MEGA 5.1 program [67] using the neighbor-joining method [68].The proteins in Solanum lycopersicum (Sl), Capsicum annuum (Ca), Arabidopsis thaliana (At), Nicotiana tabacum (Nt) and Brassica oleracea (Bo) include SlDXS1 (FN424051), SlDXS2 (FN424052), SlPSY1 (ABU40772), SlPSY2 (ABU40771), SlPDS (AGO05926), SlZ-ISO (XP_004252966), SlZDS (AGO05927), SlCRTISO (AAL91366), SlLCY-B (NP_001234226), SlCYC-B (AAG21133),SlLCY-E (Y14387), SlCRTR-B1 (CAB55625), SlCRTR-B2 (CAB55626), SlCYP97A29 (ACJ25969), SlCYP97C11 (ACJ25968), SlZEP (P93236), SlVDE (ACM92036), SlNCED1 (CAB10168), SlNCED6 (XP_004240215), SlCCD1A (AAT68187), SlCCD1B (AAT68188), SlCCD4 (XP_004246004), SlCHRC (ABC42191), SlHSP21 (AAB07023); CaTKT2 (CAA75778), CaPSY (P37272), CaPDS (P80093), CaZDS (Q9SMJ3), CaLCY-B (ADH04271), CaCCS (CAA54495), CaCRTR-B1 (CAA70888), CaCRTR-B2 (CAA70427), CaZEP (Q96375), CaFBN (CAA50750); AtCLA1 (AAC49368), AtPSY (AAM62787), AtPDS (AAL15300), AtZ-ISO (NP_001117264), AtZDS (AAM63349), AtCRTISO (NP_172167), AtLCY-B (AAB53337), AtLCY-E (AAB53336), AtCRTR-B2 (NP_200070), AtCRTR-B1 (AAC49443), AtCYP97A3 (AAL08302), AtCYP97C1 (AAM13903), AtZEP (BAB08942), AtVDE (AAC50032), AtNCED2 (AT4G18350), AtNCED3 (AT3G14440), AtNCED5 (AT1G30100), AtNCED6 (AT3G24220), AtNCED9 (AT1G78390), AtCCD1 (AT3G63520), AtCCD4 (AT4G19170), AtCCD7 (AT2G44990), AtCCD8 (AT4G32810), AtFBN (NP_192311), AtHSP21 (NP_194497); NtOr (AEV23056) and BoOr (ABH07405). [file 12870_2014_269_MOESM4_ESM.pdf]

Additional File 4 - Phylogenetic trees for the carotenogenesis-ralated proteins from *L. barbarum* (Lb), *L. ruthenicum* (Lr) and other organisms.

(a)

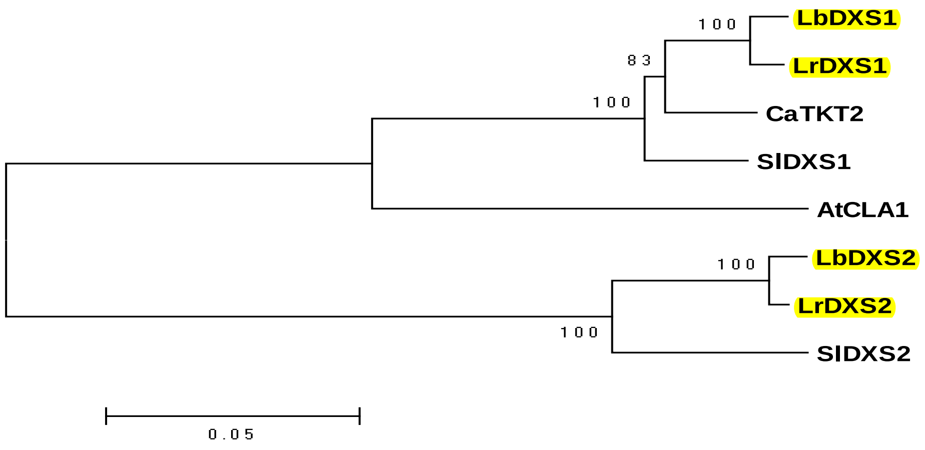

(b)

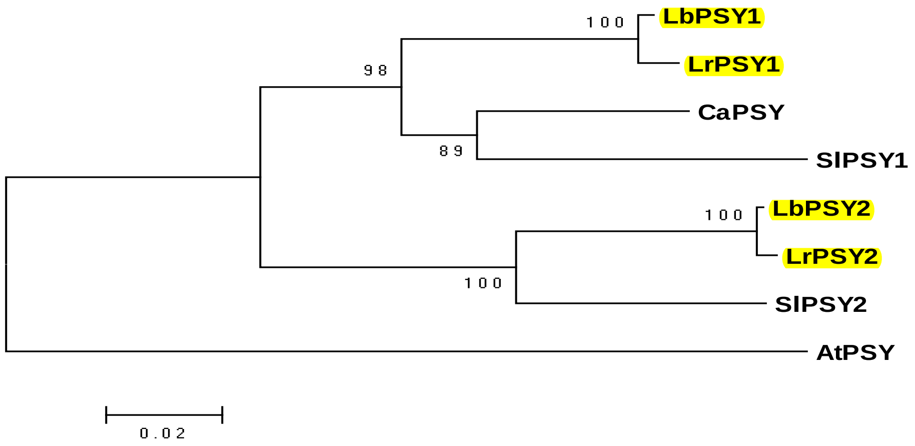

(c)

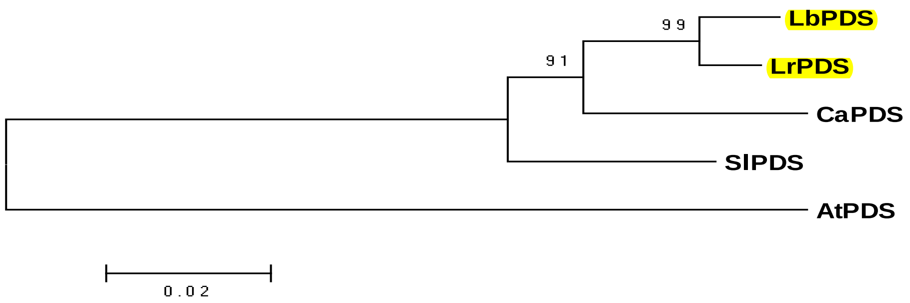

(d)

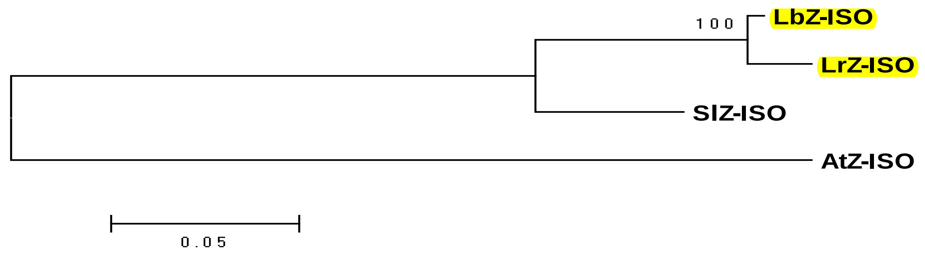
100
LbZ-ISO
LrZ-ISO
SlZ-ISO
AtZ-ISO
0.05

(e)

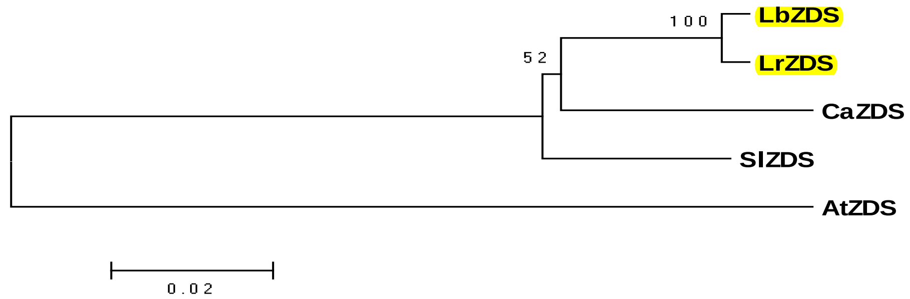
100
52
LbZDS
LrZDS
CaZDS
SlZDS
AtZDS
0.02

(f)

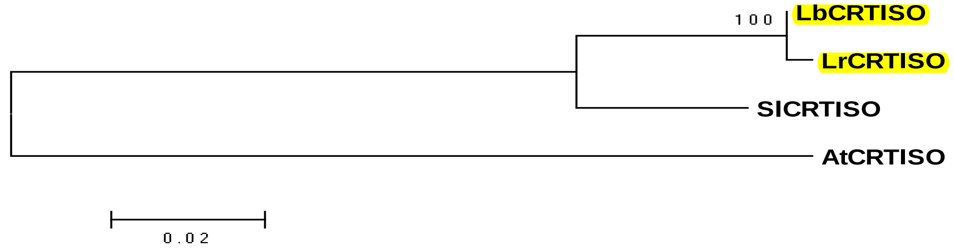
100
LbCRTISO
LrCRTISO
SlCRTISO
AtCRTISO
0.02

(g)

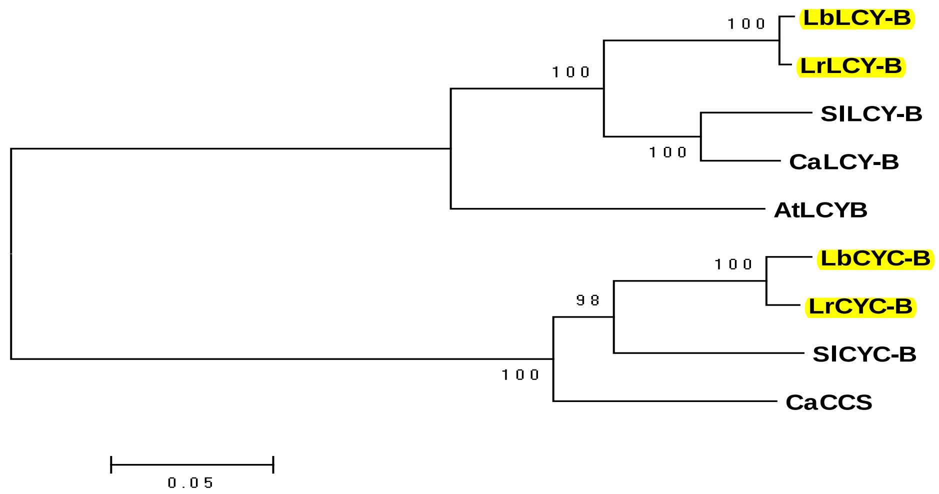
100
LbLCY-B
100
LrLCY-B
SlLCY-B
100
CaLCY-B
AtLCYB
100
LbCYC-B
98
LrCYC-B
SlCYC-B
100
CaCCS
0.05

(h)

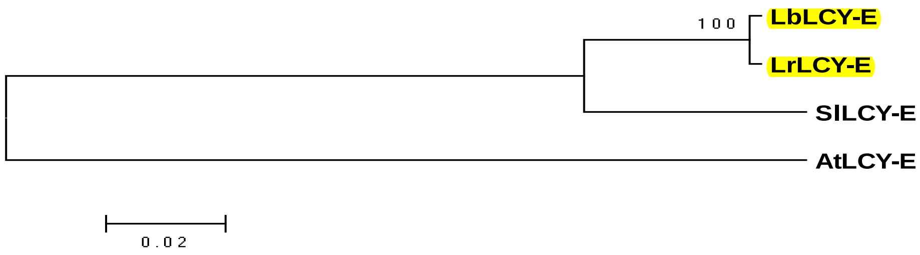
100
LbLCY-E
LrLCY-E
SlLCY-E
AtLCY-E
0.02

(i)

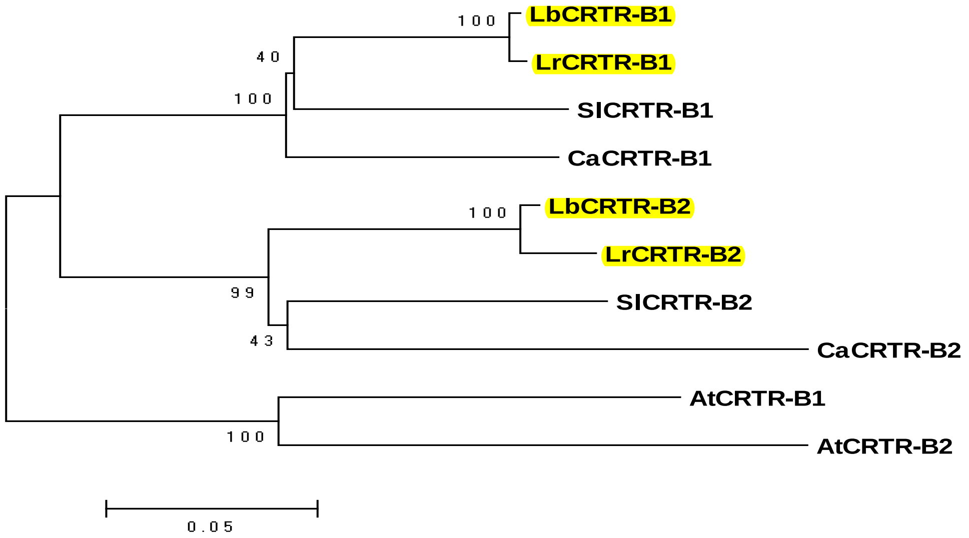
100
LbCRTR-B1
40
LrCRTR-B1
100
SlCRTR-B1
CaCRTR-B1
100
LbCRTR-B2
LrCRTR-B2
99
SlCRTR-B2
43
CaCRTR-B2
AtCRTR-B1
100
AtCRTR-B2
0.05

(j)

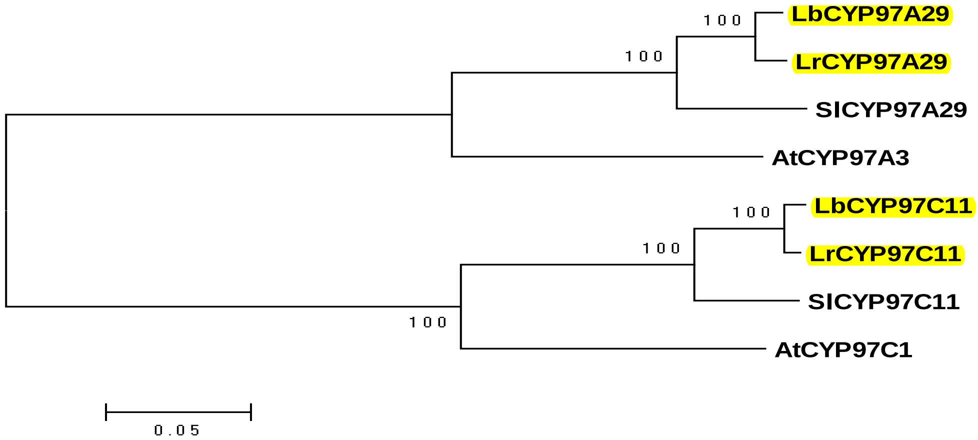
100
LbCYP97A29
100
LrCYP97A29
SlCYP97A29
AtCYP97A3
100
LbCYP97C11
100
LrCYP97C11
SlCYP97C11
100
AtCYP97C1
0.05

(k)

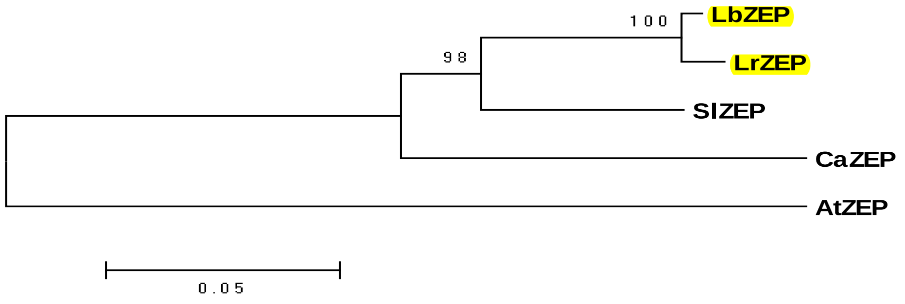
100
98
LbZEP
LrZEP
SlZEP
CaZEP
AtZEP
0.05

(l)

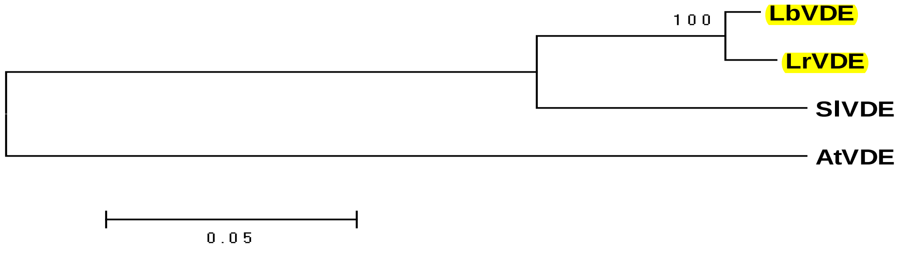
100
LbVDE
LrVDE
SlVDE
AtVDE
0.05

(m)

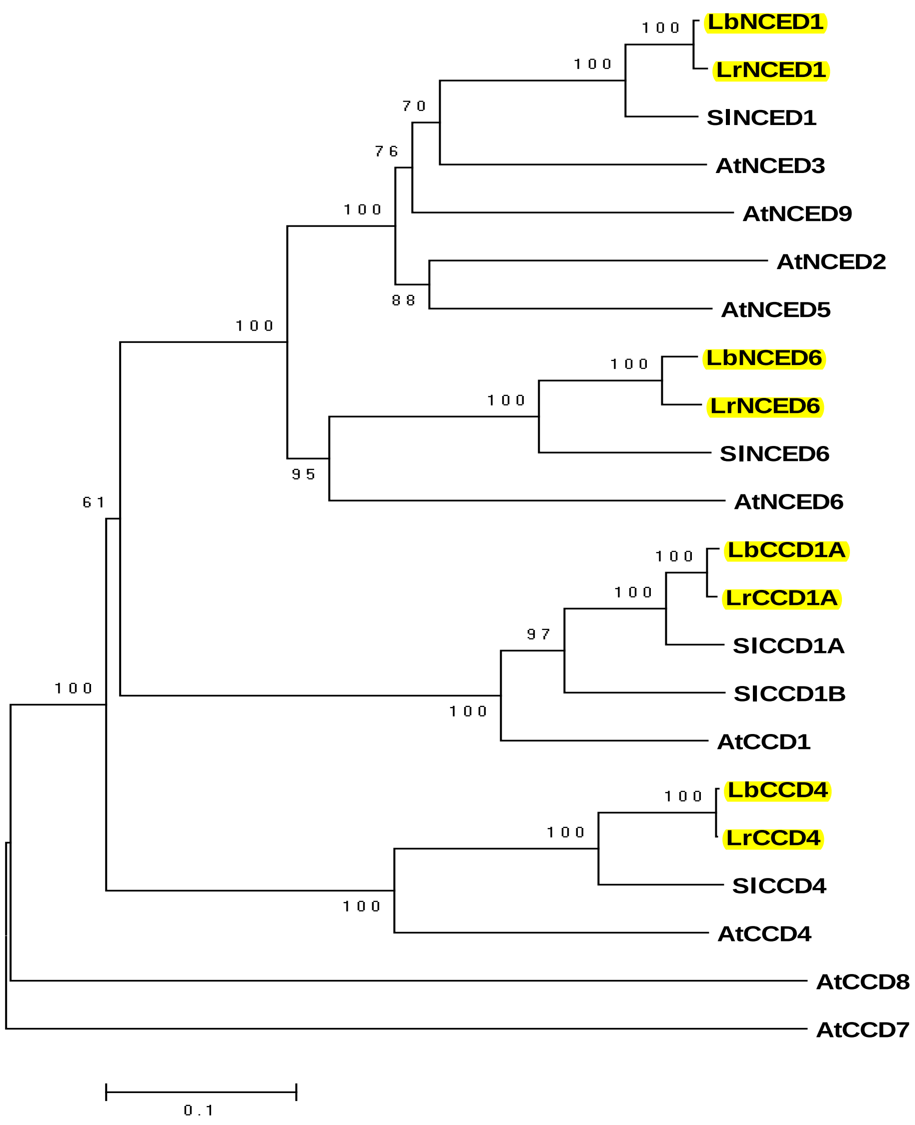
LbNCED1
LrNCED1
SlNCED1
AtNCED3
AtNCED9
AtNCED2
AtNCED5
LbNCED6
LrNCED6
SlNCED6
AtNCED6
LbCCD1A
LrCCD1A
SlCCD1A
SlCCD1B
AtCCD1
LbCCD4
LrCCD4
SlCCD4
AtCCD4
AtCCD8
AtCCD7
100
70
76
88
95
61
97
0.1

(n)

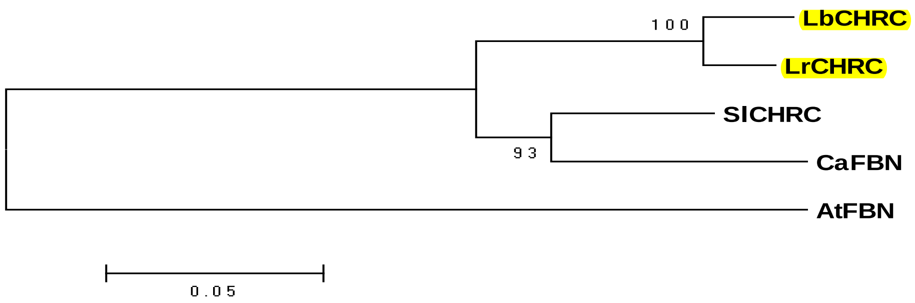
LbCHRC
LrCHRC
SlCHRC
CaFBN
AtFBN
100
93
0.05

(o)

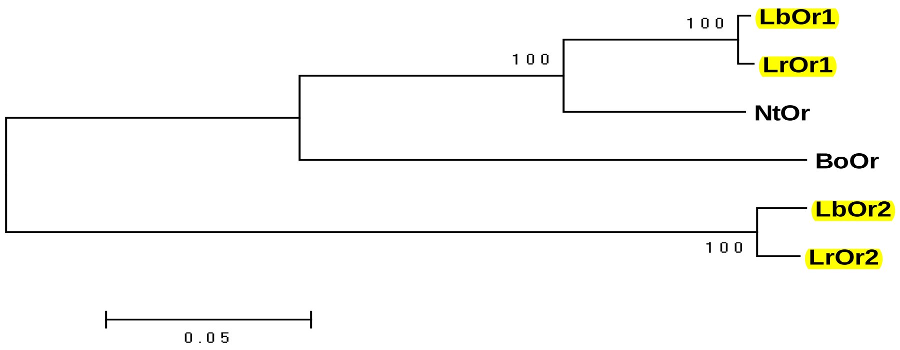
100
100
LbOr1
LrOr1
NtOr
BoOr
LbOr2
100
LrOr2
0.05

(p)

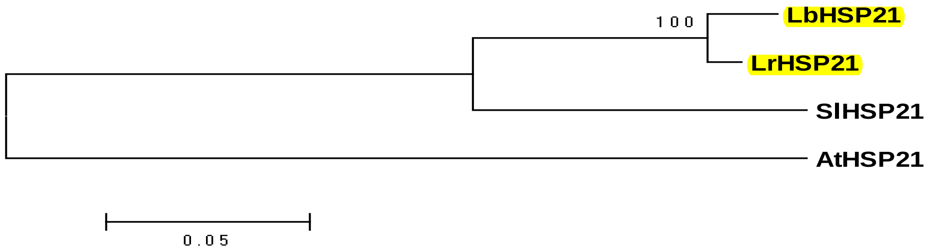
100
LbHSP21
LrHSP21
SlHSP21
AtHSP21
0.05
