# Supplementary material for: Comparative analysis of carotenoid accumulation in two goji (Lycium barbarum L. and L. ruthenicum Murr.) fruits
Source: BMC Plant Biol. 2014 Dec 16;14:269. doi: 10.1186/s12870-014-0269-4 (PMC4276078; doi:10.1186/s12870-014-0269-4)
Supplement: Additional file 9: — Specific primers used for gene cloning and qRT-PCR. [file 12870_2014_269_MOESM9_ESM.pdf]

Additional file 9 - Specific primers used for gene cloning and qRT-PCR.

Target gene	For gene clone (5'-3')	For qRT-PCR (5'-3')
LbDXS1/LrDXS1	F: ATGGCTTTGTGTGCATATGC<br>R: TTATGTCATGATCTCTAGAGCTTCTC	F: TCATCAATGGCAGCAGCAGT<br>R: AGAGCAGGTCTTGTTGGAGC
LbDXS2/LrDXS2	F: ATGGCAGTTTCTTCAGGTCC<br>R: TTAATTTACTACCATAGCTTCCTTTG	F: AGTATTCTTCCCTTACCAACCTC<br>R: GTAGCGTACATGGCTTGTTTG
LbPSY1/LrPSY1	F: ATGTCTGTTGCCTTGGCACAG<br>R: CATGCCTTAGCTTAGAGGAGAC	F: TGCTGGAGAAATGACGACGATG<br>R: GTCCAAATTTCCCGGAATAGGTATG
LbPSY2/LrPSY2	F: ATGAGCATGTCTGTTGCTTTGTTG<br>R: TCATGTCTTTGCTTGTGGAGAAG	F: ACAGGAATTCTATGTGGAAGGGGAG<br>R: CCAAACTGGACTGTGTAGAAAAACTGC
LbPDS/LrPDS	F: TGTAGGTAACTGCCAAACCAC<br>R: TGATTAAAGAATGCTAATCTTCAA	F: TGCTTGGTGGGCAATCTTATG<br>R: TGACATGGCGATGAACACCTC
LbCRTISO/LrCRTISO	F: ATGGGTACCTTGAATTTTATGTTTCC<br>R: GTCTCATGCTAGTGTCCTTAACCA	F: GAATGCCACCAGATGGGATAC<br>R: CTGGTGGGAGAACATCTGCTT
LbZDS/LrZDS	F: ATGGCTACTTCTTCAGCTTATTTTTG<br>R: TTAGACAAGACTCAACTCATCAGATAGC	F: TCAATGTGTCCTTACGCCTGG<br>R: ACTGATGACCAGGTAACCTCAAGAC
LbZ-ISO/LrZ-ISO	F: ATGGCCACTTCAATTTTTCTCTCA<br>R: CTACCAGTGTAGCCGATAACTGG	F: GGACCGAAGGTTAGCAATACG<br>R: CCTTGTAATAATCTTCGGGCAAC
LbLCY-B/LrLCY-B	F: ATGGATACACTGTTGAAAACCCC<br>R: CTCCTGATAAGTTATTCTGAATCCTG	F: TCAAGAAAGAATGGTGGCTCG<br>R: CACTCTCTGAGGTAATACCGGAAG
LbCYC-B/LrCYC-B	F: ATGGAAACTCTTCTCAGGCC<br>R: TCAAAGGCTCTCTACTGCTAG	F: TGACCCTTCACCACTTTCCA<br>R: TTCTTCATGCTTCACCTCCC
LbLCY-E/LrLCY-E	F: ATGGAGTGTGTTGGAGTTCAAA<br>R: GGCCTCTGTTCTGAAAGAGAACA	F: TGCGAGGGTGACATTGTGATTC<br>R: TTATCGACCTCAACCTCCACTCC
LbCRTR-B1/Lr CRTR-B1	F: ATGGCAGCCGCCAGAATTTCC<br>R: CCAATCATTCATGATCCCTTTG	F: TGAAGAGTTGGAAAAGGAAGTG<br>R: GTGATAGGAGTATTATCAACAATGACC
LbCRTR-B2/LrCRTR-B2	F: CTGCAAGGTGTCCACACAGTAAG<br>R: GCTAGAGCCTAATTACAGGTACATG	F: GTAGGAGGACTTGAGGAGTTGGA<br>R: GCTAGAGCCTAATTACAGGTACATG
LbCYP97A29/LrCYP97A29	F: ATGGCTGCTTCTGTTCCTCTTC<br>R: TTATGATCGAACACTAGAAGCTTCG	F: TTTCCGGTTGACTTTTGGTC<br>R:ACACGCCAAATTTCTCCATC
LbCYP97C11/LrCYP97C11	F:ATGCCATTTTCACTCACCATATCT<br>R:TTACAGAGCAGCAGCCAAAACA	F:AGTTGACAGAGTTTTGGGAGGAC<br>R:AGGACAGGTGGATGTGGATAGA
LbZEP/LrZEP	F: ATGTATTCAACTGTCTTTTACAC<br>R: TTCCCGTTTATATCAGGTCA	F: TGCTGATGGCATAAGGTCTAAGG<br>R: AACGAAGTACTGTTTGTGGCCC
LbVDE/LrVDE	F: ATGGCTCTCGCCCCTCATT<br>R: CTACCTTAATTTCCTTATTGGTAATGC	F: GCAACAATAGACCTGATGAAACGG<br>R: TTCACCCACATCAGATTTACGAGG
LbNCED1/LrNCED1	F: ATGGCCACTTCTTCTCCTGCTA<br>R: TTAGGCCTGATTTGCCAAGTCT	F:ATGATTTCGCGATAACGGAG<br>R:ACAATCAGGCACTTCAACCC
LbNCED6/LrNCED6	F: ATGTCTTTCTCATCTTCCACCGT<br>R: AACAAGAAGAAGATTGATTACATAAATC	F:CCGGTTTTTCCTAAACCGAT<br>R:TTGCATCAACCAAACCAAAA
LbCCD1A/LrCCD1A	F:ATGGGGAGAAAAGAAGATGATGA<br>R:TCACAGCTTGGCTTGTTCTTGA	F:GCTTTTGGCACTTTCAGAGG<br>R:ACGGTGGGGTGTGAGAGTAG
LbCCD4/LrCCD4	F: GCCTTACTCCTCACTCTCCTACTC<br>R: CCTGCCAATTCCATTCATCAC	F: TGCCAAAGGTAACAGGGGTAG<br>R: GAAGGAGCCTTCTCCAAATAGTC

LbCHRC/LrCHRC	F: ATGGCTTCCATCTCTTCTCTCAATC<br>R: TTAAGGCTTCAAGAGGGGACTGC	F: TCCATTTCAAATCAACCACCAA<br>R: AAACACTGCCTGCATCTCCTCT
LbOr1/LrOr1	F: ATGGTGTGGGCAGGTAGAATG<br>R: TTAATCGAAGGGATCAATTCG	F: CTGCATTGATGGTTGTTGAAATC<br>R: TGACAGAGGGTTGTCCGCTC
LbOr2/LrOr2	F: ATGGGCTCTATGTTTTTTTGC<br>R: TTAATCAAATGGATCTATACGAAGG	F: GAAGCTAACAACGTGGTCCAA<br>R: TTAAGAGATGGTACACGCAACG
LbHSP21/LrHSP21	F: TGGCAATCACCACTCCTTCA<br>R: GGAAAGGGAAACAGGAAGAGC	F: GACAGACTATTCGAAGATGCCC<br>R: ATTTTCCACGGATACTTTCACG
LbACTIN/LrACTIN		F: CAATCGGGTATTTCAAGGTCAAG<br>R: GAGCAGTGTTTCCCAGCATTG
